# Smoking habit and long-term colorectal cancer incidence by exome-wide mutational and neoantigen loads: evidence based on the prospective cohort incident-tumour biobank method

**DOI:** 10.1136/bmjonc-2025-000787

**Published:** 2025-06-03

**Authors:** Tsuyoshi Hamada, Tomotaka Ugai, Carino Gurjao, Satoko Ugai, Xuehong Zhang, Koichiro Haruki, Yasutoshi Takashima, Naohiko Akimoto, Mai Chan Lau, Kosuke Matsuda, Nobuhiro Nakazawa, Mayu Higashioka, Satoshi Miyahara, Keisuke Kosumi, Yohei Masugi, Li Liu, Yin Cao, Daniel Nevo, Molin Wang, Reiko Nishihara, Sachet A Shukla, Catherine J Wu, Levi A Garraway, Jeffrey A Meyerhardt, Edward L Giovannucci, Jonathan A Nowak, Charles S Fuchs, Andrew T Chan, Mingyang Song, Marios Giannakis, Shuji Ogino

**Affiliations:** 1 Program in MPE Molecular Pathological Epidemiology, Department of Pathology, Brigham and Women’s Hospital, Boston, Massachusetts, USA; 2 Department of Gastroenterology, Graduate School of Medicine, The University of Tokyo, Tokyo, Japan; 3 Department of Epidemiology, Harvard T.H. Chan School of Public Health, Boston, Massachusetts, USA; 4 Department of Medical Oncology, Dana-Farber Cancer Institute, Boston, Massachusetts, USA; 5 Broad Institute of MIT and Harvard, Cambridge, Massachusetts, USA; 6 Department of Nutrition, Harvard T.H. Chan School of Public Health, Boston, Massachusetts, USA; 7 Yale University School of Nursing, Orange, Connecticut, USA; 8 Department of Epidemiology and Biostatistics, and the Ministry of Education Key Lab of Environment and Health, Huazhong University of Science and Technology, Hubei, China; 9 Division of Public Health Sciences, Department of Surgery, Washington University School of Medicine, St. Louis, Missouri, USA; 10 Clinical and Translational Epidemiology Unit, Massachusetts General Hospital, Boston, Massachusetts, USA; 11 Department of Biostatistics, Harvard T.H. Chan School of Public Health, Boston, Massachusetts, USA; 12 Channing Division of Network Medicine, Department of Medicine, Brigham and Women’s Hospital, Boston, Massachusetts, USA; 13 Department of Hematopoietic Biology and Malignancy, The University of Texas MD Anderson Cancer Center, Houston, Texas, USA; 14 Department of Medicine, Brigham and Women’s Hospital, Boston, Massachusetts, USA; 15 Genentech Inc, South San Francisco, California, USA; 16 Division of Gastroenterology, Massachusetts General Hospital, Boston, Massachusetts, USA; 17 Department of Immunology and Infectious Diseases, Harvard T.H. Chan School of Public Health, Boston, Massachusetts, USA; 18 Cancer Immunology and Cancer Epidemiology Programs, Dana-Farber/Harvard Cancer Center, Boston, Massachusetts, USA; 19 Institute of Science Tokyo, Tokyo, Japan

**Keywords:** Colorectal cancer, Epidemiology, Genetic markers, Immunogenicity, Tumour biomarkers

## Abstract

**Objective:**

To test the hypothesis that the association of smoking with long-term colorectal cancer incidence may be stronger for tumours with higher mutational and neoantigen loads.

**Methods and analysis:**

In the Nurses’ Health Study (1980–2012) and the Health Professionals Follow-up Study (1986–2012), our novel prospective cohort incident-tumour biobank method (PCIBM) used 3053 incident colorectal carcinoma cases including 752 cases with whole-exome sequencing data. Using the multivariable duplication-method Cox regression model with the inverse probability weighting to adjust for the selection bias due to tissue availability, we assessed a differential association of cigarette smoking with colorectal carcinoma incidence by an exome-wide tumour mutational burden (e-TMB) or neoantigen load.

**Results:**

The association of pack-years smoked with colorectal cancer incidence differed by e-TMB (P_heterogeneity_<0.001). Multivariable-adjusted HRs for e-TMB-high (≥10 mutations/megabase) tumours were 1.28 (95% CI 0.72 to 2.28) and 2.56 (95% CI 1.61 to 4.07) for 1–19 and ≥20 pack-years (vs 0 pack-years; P_trend_<0.001), respectively. In contrast, pack-years smoked were not associated with e-TMB-low tumour incidence (P_trend_=0.67). A similar differential association was observed for the neoantigen load (P_heterogeneity_=0.017). The differential association by e-TMB appeared consistent in the strata of CpG island methylator phenotype status, *BRAF* mutation or lymphocytic infiltrates.

**Conclusions:**

Smoking is more strongly associated with the long-term incidence of colorectal carcinoma harbouring higher mutational and neoantigen loads. Our PCIBM-based evidence supports the immunosuppressive effect of smoking and the potential of smoking cessation in improving antitumour immunity for cancer prevention and treatment.

**WHAT IS ALREADY KNOWN ON THIS TOPIC**

**WHAT THIS STUDY ADDS**

- Using the prospective cohort incident-tumour biobank method (PCIBM), we found a stronger association of smoking with the incidence of colorectal cancer harbouring higher exome-wide mutation and neoantigen loads.
- Our findings provide unique evidence for the interplay of smoking and tumour somatic mutations (likely influencing antitumour immune response) during tumour development.

**HOW THIS STUDY MIGHT AFFECT RESEARCH, PRACTICE OR POLICY**

- Our PCIBM-based study supports the role of smoking cessation as an immune-enhancing intervention for cancer-free persons and patients with colorectal cancer.
- Further research is warranted to examine the synergistic effect of smoking cessation and the immune checkpoint blockade in patients with cancer.

## Introduction

 Immunotherapy has become a major therapeutic modality in clinical oncology. Microsatellite instability (MSI)-high status currently serves as a reliable clinical biomarker for response to the immune checkpoint blockade in solid tumours.^1^,^5^ The therapeutic responsiveness of MSI-high tumours may be attributable to an increased abundance of immunogenic neopeptides (‘neoantigens’), some of which directly stimulate the immune response.^6^,^9^ In addition, tumour mutation burden (TMB, typically measured as the number of nonsynonymous mutations per megabase sequenced) can be used as another tumour biomarker separate from the MSI status.^10 11^ Identifying nonsynonymous somatic mutations (and resulting neoantigens) throughout the exome requires data from whole exome, genome or transcriptome sequencing on a pair of tumour and normal specimens. As such, omics sequencing technologies are increasingly utilised in clinical practice. Exome-wide TMB (referred to as ‘e-TMB’ hereafter) and neoantigens (rather than MSI status) will be clinical biomarkers for response to immunotherapy. For tumour immunogenicity measurements, e-TMB is superior to commonly used TMB based on targeted sequencing assays of selected cancer-associated genes because gain or loss of function driver mutations in those cancer-associated genes have high selection pressures during tumour evolution.^10^,^12^

Cigarette smoking is a modest risk factor for colorectal carcinomas.^13^,^16^ Although cigarette smoke is a known mutagen linked to the ‘smoking mutational signature,’^17 18^ this signature has not been observed in colorectal carcinoma. Epidemiological studies have consistently shown a stronger association of smoking with colorectal cancer incidence for MSI-high tumours than for non-MSI-high tumours.^13^,^16^ There appears to be an even stronger association of smoking with MSI-high colorectal carcinoma containing fewer T cells, supporting the immunosuppressive effects of smoking that likely play a pathogenic role in MSI-high colorectal tumours.^19^ Considering that neoantigens (rather than MSI-high status per se) directly stimulate antitumour immune response, we hypothesised that the association of smoking with long-term colorectal cancer incidence might be stronger for tumours with higher levels of e-TMB and neoantigen loads.

Using two large prospective cohort studies in the USA with data on long-term smoking habit and whole-exome sequencing (WES) of colorectal cancer and matched normal tissue, we examined longitudinally updated pack-years smoked and the long-term incidence of colorectal carcinomas subclassified by e-TMB or neoantigen loads. We applied the prospective cohort incident-tumour biobank method (PCIBM)^20 21^ to decades-long prospective collection of comprehensive lifestyle data and tumour genomic profiling. The current study represents the first prospective investigation of longitudinally updated smoking habit in relation to the long-term incidence of colorectal carcinomas classified by somatic genomic profiles based on WES.

## Materials and methods

### Study population

**Figure 1:**
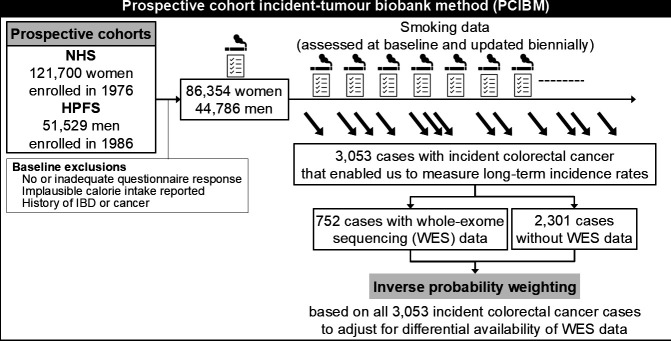
Flow diagram of the study population in the Nurses’ Health Study (NHS) and the Health Professionals Follow-up Study (HPFS). In the current study based on the PCIBM, we included 86 354 women and 44 786 men who were followed for decades and examined the incidence of colorectal carcinomas subclassified by exome-wide tumour mutational burden or neoantigen loads. To reduce selection bias due to tissue availability, we applied the inverse probability weighting for 752 cases with available WES data using the data from 3053 cases. IBD, inflammatory bowel disease.

We used the PCIBM^20 21^ on data from two ongoing prospective cohort studies in the USA, the Nurses’ Health Study (NHS, 121 700 women aged 30–55 years followed since 1976) and the Health Professionals Follow-up Study (HPFS, 51 529 men aged 40–75 years followed since 1986) (table 1 and figure 1).^22 23^ Using mailed biennial questionnaires, participants have reported lifestyle factors including smoking behaviour and newly diagnosed diseases. The response rate has exceeded 90% for each follow-up questionnaire cycle in both cohorts. At the baseline (1980 for the NHS and 1986 for the HPFS), we excluded participants who did not return the initial food frequency questionnaire, left a large number of items blank (>10 of 61 items for the NHS and >70 of 131 items for the HPFS), reported unreasonable total calorie intake (<600 or >3500 calories/day for women, and <800 or >4200 calories/day for men), or reported a history of inflammatory bowel disease. We additionally excluded participants with a history of cancer except for non-melanoma skin cancer to rule out the possibility of metastatic tumours to the colorectum and that of biases derived from lifestyle alterations due to cancer diagnosis. Participants were followed until death or the end of follow-up (1 June 2012 for the NHS; and 31 January 2012 for the HPFS), whichever came first.

**Table 1:** Age-standardised characteristics of participants according to cumulative pack-years smoked in the Nurses’ Health Study (NHS, 1980–2012) and the Health Professionals Follow-up Study (HPFS, 1986–2012)

	Women (NHS)			Men (HPFS)		
	Cumulative pack-years smoked			Cumulative pack-years smoked		
Characteristic*	0	1–19	≥20	0	1–19	≥20
Person-years	1 125 146	705 774	661 296	483 866	231 160	266 200
Age, years	60.9 (11.5)	59.4 (11.5)	61.6 (10.7)	63.2 (11.3)	63.3 (11.1)	66.6 (10.6)
Family history of colorectal cancer	13%	14%	13%	13%	12%	12%
History of diabetes	7.4%	6.8%	7.8%	6.8%	7.3%	9.3%
Body mass index, kg/m²	25.5 (4.7)	25.2 (4.6)	25.1 (4.5)	25.6 (3.4)	25.7 (3.2)	26.3 (3.6)
Postmenopause	76%	76%	82%	–	–	–
Menopausal hormone therapy	28%	29%	24%	–	–	–
History of colonoscopy/sigmoidoscopy	40%	43%	36%	54%	56%	50%
Regular use of multivitamins	53%	54%	48%	44%	45%	43%
Regular use of aspirin	39%	40%	41%	46%	49%	49%
Regular use of other NSAIDs	17%	19%	18%	15%	17%	16%
Physical activity, METS-hours/week	16.5 (16.8)	18.0 (18.8)	15.1 (16.3)	26.9 (23.6)	27.0 (22.6)	22.3 (21.1)
Total calorie intake, kcal/day	1702 (443)	1677 (432)	1645 (439)	1983 (554)	1965 (550)	1980 (560)
Alcohol intake, g/day	3.8 (6.9)	6.9 (8.9)	8.7 (11.8)	8.0 (11.1)	12.5 (13.8)	15.1 (16.9)
Red and processed meat intake, servings/week	6.6 (3.7)	6.3 (3.5)	6.8 (3.7)	6.1 (4.3)	6.1 (4.2)	7.2 (4.8)
Total calcium intake, mg/day	939 (357)	953 (352)	887 (350)	957 (375)	932 (367)	897 (374)
Total folate intake, μg/day	429 (212)	439 (212)	398 (204)	552 (253)	560 (257)	511 (250)
Alternate Healthy Eating Index 2010†	46.1 (9.6)	47.5 (9.6)	45.3 (9.6)	48.6 (10.1)	49.0 (9.9)	46.6 (10.1)

* All variables other than age were standardised to age distribution of each cohort. Mean (SD) was presented for continuous variables.

† Without alcohol intake.

METS, Metabolic Equivalent Task Score; NSAID, non-steroidal anti-inflammatory drug.

### Assessment of smoking behaviour

The details of smoking behaviour were assessed as reported previously.^19 24^ In 1976 (the NHS) and 1986 (the HPFS), participants reported the age when they began smoking (and ceased smoking, if applicable), as well as the average daily consumption of cigarettes. Participants have updated current smoking status and daily cigarette consumption every 2 years. We calculated cumulative pack-years smoked (average daily consumption of cigarette packs multiplied by the number of years smoked) at the baseline and every 2 years thereafter.

### Acquisition of colorectal cancer cases

In both cohorts, colorectal carcinoma cases were identified based on biennial questionnaires. For non-respondents, colorectal cancer cases and deaths were ascertained through family members, US post office authorities and/or the National Death Index. Study physicians, blinded to exposure data, reviewed medical records of identified colorectal cancer cases to confirm the diagnosis and record tumour characteristics (eg*,* anatomical location and disease stage). We included both colon and rectal carcinomas based on the colorectal continuum model.^25^ We collected formalin-fixed paraffin-embedded (FFPE) tissue blocks of surgically resected colorectal tumours from hospitals throughout the USA, and the study pathologist (SO) confirmed a pathological diagnosis of colorectal carcinoma. During the follow-up of the participants, we documented 3053 incident colorectal cancer cases, including 752 cases with available WES data. There were no substantial differences in clinical data between cases with and without WES data (online supplemental table S1).

### Analyses of colorectal cancer tissue

The study pathologist (SO), blinded to other data, reviewed H&E-stained tissue sections and recorded pathological features including four patterns of lymphocytic reaction (tumour-infiltrating lymphocytes, intratumoural periglandular reaction, peritumoural lymphocytic reaction and Crohn’s-like lymphoid reaction).^26 27^ Tumour status of MSI, CIMP and *BRAF* mutation was assessed as previously described.^25 28 29^ Tumour MSI status was assessed using PCR of 10 microsatellite markers (D2S123, D5S346, D17S250, BAT25, BAT26, BAT40, D18S55, D18S56, D18S67 and D18S487), and MSI-high was defined as the presence of instability in ≥30% of the markers.^25^

### WES and downstream analyses

The study pathologist (SO) marked tumour areas in guide H&E-stained slides. Using the guide H&E slides to ensure high tumour cellularity, DNA was extracted from tumour areas in sections of archival FFPE blocks.^30^ Matched normal DNA was obtained from normal colon tissue that was grossly away from the tumour. As previously described,^31^ we performed WES on DNA from tumour and matched normal tissue pairs with the mean target coverage of 85× and the mean of 49 million paired-end reads across all samples. To remove artefacts resulting from hydrolytic deamination of cytosine to uracil in FFPE samples, we filtered out C to T transition mutations as possible FFPE-specific artefacts using the tool described elsewhere.^32^ To further filter out spurious single-nucleotide variant calls, we used BWA (Burrows-Wheeler Aligner)-MEM (http://bio-bwa.sourceforge.net/) to realign sequenced reads associated with the mutations to a set of sequences derived from the human reference assembly. e-TMB was defined as the number of non-synonymous mutations per megabase covered in the whole exome. We also calculated TMB based on the panel of selected cancer-associated genes (called ‘targeted TMB’), that is, 447 genes used in clinical practice at the Brigham and Women’s Hospital (listed in online supplemental table S2). The neoantigen load (ie, the number of mutated proteins that likely give rise to immunogenic peptides) was calculated by counting peptides that were predicted to bind to HLA molecules with high affinity (the rank <0.5%), as previously described.^33^ Using NetMHCpan (V.4.1),^33^ we predicted the binding affinities of 9-mer and 10-mer mutant peptides found in tumours to the corresponding *HLA* alleles inferred by the POLYSOLVER algorithm.^34^ e-TMB was categorised as high and low at the cut-off point of 10 mutations per megabase that was adopted for the US Food and Drug Administration approval of pembrolizumab for TMB-high tumours and has been commonly used.^35 36^ Given that TMB-high cases represented 13% of all cases with available WES data, the neoantigen load was categorised as high (≥326 per exome, the top quartile) and low (<326 per exome, the other quartiles). We examined the single-base substitution (SBS) signatures,^17^ including SBS11 (alkylating signature) associated with red meat consumption in individuals developing colorectal cancer.^31^ SBS4 (smoking signature) characterised by C–A nucleotide transversions was specifically examined.

### Statistical analysis

All statistical analyses were performed using SAS software (V.9.4, SAS Institute), and all p values were two-sided. We used the two-sided α level of 0.005, as recommended by expert statisticians.^37^ Our primary hypothesis testing was an assessment of the heterogeneity between associations of cumulative pack-years smoked (continuous with a ceiling at 50 pack-years) with the incidence of colorectal cancer subclassified by e-TMB or neoantigen loads (high vs low).

We used the Cox proportional hazards regression model to estimate the HR for colorectal cancer incidence. To assess differential associations of smoking variables with the incidence of colorectal cancer subclassified by e-TMB or neoantigen loads, we used the duplication-method Cox regression model for competing risks.^38^ Using the likelihood ratio test (1 df), we examined a heterogeneity trend across tumour subtypes in a statistical trend of the association across smoking exposure levels.^39^ The multivariable Cox regression model included the covariates described in table 2. We treated all exposure variables as time-dependent to account for changes over time. To reduce intraindividual variation and consider long-term influences, we used the cumulative average for relevant variables, which was the mean of all available data prior to each questionnaire cycle. The Cox regression models were stratified by sex (for pooled analyses), age and calendar year of the questionnaire cycle. Colorectal cancer cases without WES data were treated as censored at the time of cancer diagnosis.

**Table 2:** Cumulative pack-years smoked and colorectal cancer incidence, overall and by exome-wide tumour mutational burden or neoantigen loads

	Cumulative pack-years smoked				
	0	1–19	≥20	P_trend_*	P_heterogeneity_†
Person-years	1 609 012	936 934	927 496		
All colorectal cancer (n=752)					
n	316	189	247		
Age-adjusted HR (95% CI)‡	1 (referent)	1.12 (0.94 to 1.35)	1.20 (1.02 to 1.43)	0.012	–
Multivariable HR (95% CI)‡§	1 (referent)	1.15 (0.95 to 1.38)	1.16 (0.97 to 1.37)	0.080	–
Exome-wide tumour mutational burden¶					<0.001
Low (n=654)					
n	286	169	199		
Age-adjusted HR (95% CI)‡	1 (referent)	1.11 (0.92 to 1.34)	1.08 (0.90 to 1.29)	0.36	
Multivariable HR (95% CI)‡§	1 (referent)	1.14 (0.94 to 1.38)	1.05 (0.87 to 1.27)	0.67	
High (n=98)					
n	30	20	48		
Age-adjusted HR (95% CI)‡	1 (referent)	1.24 (0.69 to 2.22)	2.61 (1.64 to 4.15)	<0.001	
Multivariable HR (95% CI)‡§	1 (referent)	1.28 (0.72 to 2.28)	2.56 (1.61 to 4.07)	<0.001	
Neoantigen loads¶					0.017
Low (n=564)					
n	242	154	168		
Age-adjusted HR (95% CI)‡	1 (referent)	1.19 (0.97 to 1.45)	1.06 (0.87 to 1.30)	0.34	
Multivariable HR (95% CI)‡§	1 (referent)	1.22 (0.99 to 1.49)	1.03 (0.84 to 1.27)	0.62	
High (n=188)					
n	74	35	79		
Age-adjusted HR (95% CI)‡	1 (referent)	0.91 (0.60 to 1.36)	1.76 (1.27 to 2.42)	<0.001	
Multivariable HR (95% CI)‡§	1 (referent)	0.95 (0.63 to 1.42)	1.73 (1.25 to 2.39)	0.002	

* P_trend_ was calculated using a linear trend test and cumulative pack-years smoked (continuous with a ceiling at 50 pack-years).

† P_heterogeneity_ was calculated using the likelihood ratio test (1 df) for the heterogeneity of binary subtype-specific associations of cumulative pack-years smoked (continuous with a ceiling at 50 pack-years) in multivariable models.

‡ Inverse probability weighting was applied to reduce a potential selection bias due to the differential availability of whole-exome sequencing data (see ‘Statistical analysis’ subsection for details).

§ The multivariable Cox regression model included family history of colorectal cancer (present vs absent), body mass index (continuous with a ceiling at 35 kg/m2), history of colonoscopy/sigmoidoscopy (present vs absent), use of aspirin or other non-steroidal anti-inflammatory drugs (regular use vs non-use), physical activity (continuous with a ceiling at 50 metabolic equivalent task score-hours/week), alcohol intake (continuous with a ceiling at 30 g/day), red and processed meat intake (continuous with a ceiling at 14 servings/week) and total folate intake (continuous with a ceiling at 1000 µg/day). For women, we additionally included menopause status/menopausal hormone therapy (premenopause vs postmenopause with never, past or current use of menopausal hormone therapy).

¶ e-TMB was categorised into high (≥10 per megabase) and low (<10 per megabase). Based on all colorectal cancer cases with available whole-exome sequencing data, neoantigen loads were categorised into high (≥326 per exome, the top quartile) and low (<326 per exome, the other quartiles).

e-TMB, exome-wide tumour mutational burden.

To adjust for selection bias caused by the availability of WES data, we used the inverse probability weighting (IPW) method (figure 1).^40^ Using covariate data on the 3053 incident colorectal cancer cases, we constructed the multivariable logistic regression model to calculate the cohort-specific probability of WES data availability in each patient. In the IPW-adjusted Cox regression model, each colorectal cancer patient with available WES data was weighted by the inverse of the probability. For example, when a patient with colorectal cancer with available WES data was estimated (based on the patient’s statuses of covariates) to have the data with a probability of 0.8, this patient was weighted by the inverse probability (ie, 1/0.8=1.25). Through this statistical approach, a bias due to the differential availability of WES data according to the covariate statuses was mitigated, increasing the representativeness of our sample of colorectal cancer cases and enhancing the generalisability of our results. Weights greater than the 95th percentile were truncated and set to the 95th percentile to reduce outlier effects. We confirmed that results with and without weight truncation did not differ substantially (data not shown).

We conducted tests of heterogeneity between the two cohorts using the *Q* statistic and observed no statistically significant heterogeneity (P_heterogeneity_>0.009) in the associations between cumulative pack-years smoked and the incidence of colorectal cancer subclassified by e-TMB or neoantigen loads. We, therefore, combined the cohorts with the adjustment for cohort (ie, sex) for further analyses to increase statistical power.

### Patient and public involvement

Patients and/or the public were not involved in the design, conduct, reporting or dissemination plans of this research.

## Results

Table 1 summarises age-standardised characteristics of the cohort participants (the histograms of cumulative pack-years smoked are presented in online supplemental figure S1). During 3 473 441 person-years follow-up of 131 140 participants, we documented 3053 colorectal cancer cases including 752 cases with available WES data (online supplemental figure S2), which yielded e-TMB (non-synonymous mutation count per megabase; median, 1.6; IQR, 1.0–3.5 and total range, 0–152.0) and neoantigen loads (median, 202; IQR, 137–326 and total range, 0–11,110). Tumour MSI status, e-TMB and neoantigen loads correlated with each other (online supplemental figure S3). Cumulative pack-years smoked were not associated with the smoking signature (online supplemental figure S4). The levels of e-TMB and neoantigen loads were not correlated with the smoking signature (online supplemental figure S5). Cumulative pack-years smoked were associated with the incidence of colorectal cancer using all of the incident cases, and this association appeared persistent regardless of WES data availability (online supplemental table S3). For further incidence analyses (except for a sensitivity analysis), we used the IPW method and the 3053 cases to adjust for selection bias due to tissue data availability.

**Figure 2:**
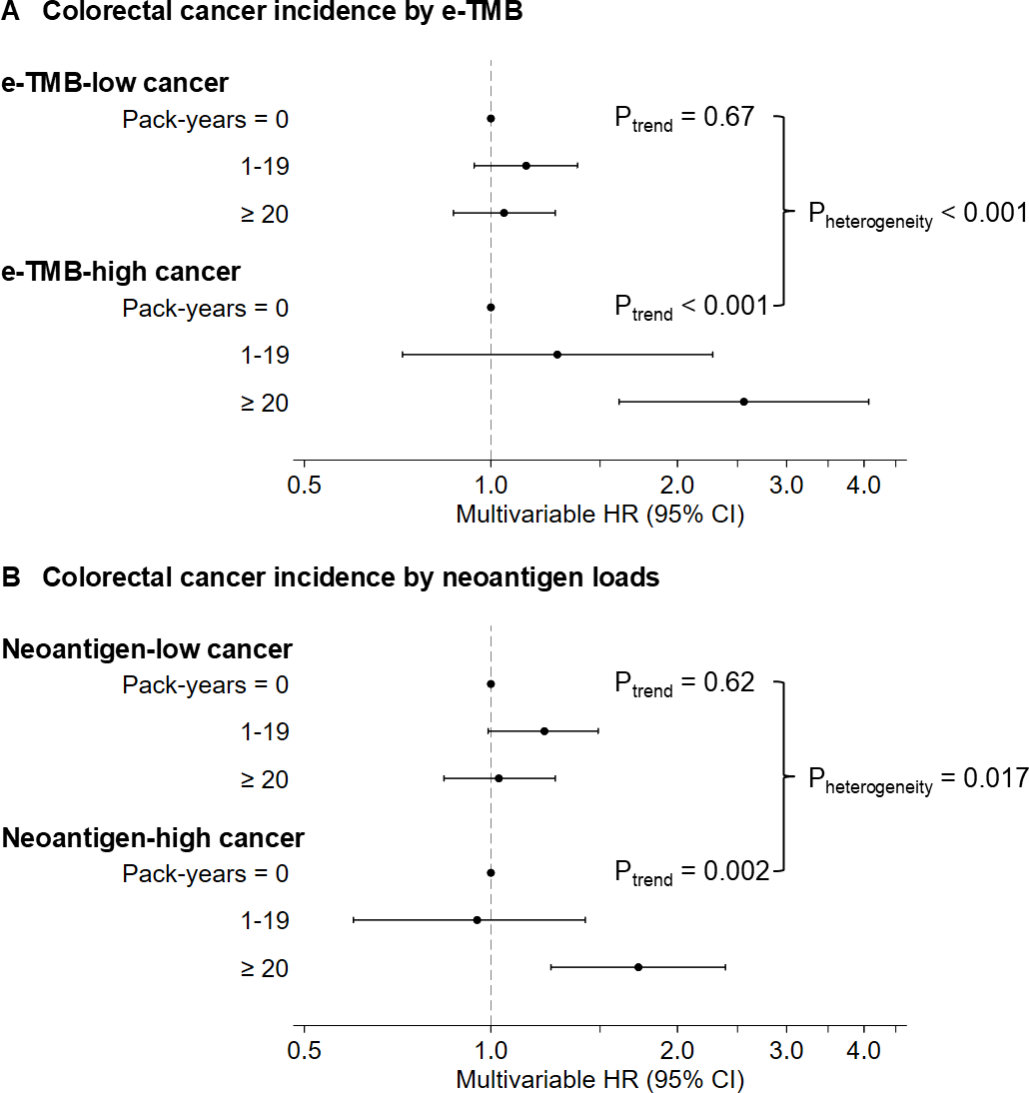
Forest plot of multivariable HRs for the incidence of colorectal cancer classified by exome-wide tumour mutational burden (**A**) or neoantigen loads (**B**) according to cumulative pack-years smoked. The dot indicates a stratum-specific multivariable HR, and the horizontal bar indicates 95% CI. The HRs were adjusted for the same set of covariates as table 2, and inverse probability weighting was applied to reduce a potential selection bias due to the differential availability of whole-exome sequencing data (see ‘Statistical analysis’ subsection for details). P_trend_ was calculated using a linear trend test and cumulative pack-years smoked (continuous with a ceiling at 50 pack-years). P_heterogeneity_ was calculated using the likelihood ratio test (1 df) for the ordinal trend heterogeneity of quartile subtype-specific associations of cumulative pack-years smoked (continuous with a ceiling at 50 pack-years). e-TMB, exome-wide tumour mutational burden.

In our primary hypothesis testing, the association of cumulative pack-years smoked with colorectal cancer incidence differed by e-TMB (P_heterogeneity_<0.001, table 2 and figure 2). Compared with never smokers, multivariable-adjusted HRs for TMB-high colorectal cancer in individuals who smoked 1–19 and ≥20 pack-years were 1.28 (95% CI 0.72 to 2.28) and 2.56 (95% CI, 1.61 to 4.07), respectively (P_trend_<0.001). In contrast, cumulative pack-years smoked were not significantly associated with the incidence of colorectal cancer containing lower levels of e-TMB (P_trend_=0.67). A similar differential association was observed for neoantigen loads (P_heterogeneity_=0.017, table 2). Our findings in each cohort are presented in online supplemental table S4. Our sensitivity analyses without IPW adjustment yielded similar results to those with IPW adjustment (online supplemental table S5). As targeted sequencing of selected cancer-associated genes has become common in clinical practice, TMB measures calculated from such targeted sequencing analyses are increasingly used. Thus, we calculated ‘targeted TMB’ based on a clinical panel of cancer-associated genes used in the Brigham and Women’s Hospital, which was only moderately correlated with e-TMB (correlation coefficient, 0.62; online supplemental figure S6). We conducted analyses using ‘targeted TMB’ and found similar but attenuated results compared with those using e-TMB (online supplemental table S6).

In secondary analyses, we examined smoking status (current vs past vs never) and duration of smoking cessation in relation to the incidence of colorectal carcinomas subclassified by e-TMB or neoantigen loads. Compared with never smoking, former and current smoking was associated with a higher incidence of colorectal cancer harbouring high levels of e-TMB but not with colorectal cancer harbouring lower levels of e-TMB (P_heterogeneity_=0.003, online supplemental table S7). Similarly, an association of the duration of smoking cessation with the incidence of colorectal carcinomas was stronger for carcinomas harbouring high levels of e-TMB (P_heterogeneity_=0.001, online supplemental table S8).

We conducted secondary subgroup analyses. In an analysis stratified by tumour MSI status, we observed a stronger association of cumulative pack-years smoked with the incidence of tumours containing higher levels of e-TMB or neoantigen loads in the stratum of MSI-high tumours (table 3), though the differential associations did not reach statistical significance. Our previous analyses suggest that smoking status may be differentially associated with the incidence of colorectal cancer by tumour status of CIMP, *BRAF* mutation and T lymphocyte infiltrates.^14 19^ Therefore, we conducted additional analyses stratified by tumour status of CIMP, *BRAF* mutation or lymphocytic reaction, which yielded similar differential associations, though statistical power was limited in the respective strata (online supplemental table S9).

**Table 3:** Cumulative pack-years smoked and colorectal cancer incidence by exome-wide tumour mutational burden or neoantigen loads in the strata of tumour microsatellite instability (MSI) status

	Cumulative pack-years smoked				
	0	1–19	≥20	P_trend_*	P_heterogeneity_†
MSI-high					
Exome-wide tumour mutational burden‡					0.14
Low (n=61)					
n	20	15	26		
Age-adjusted HR (95% CI)§	1 (referent)	1.35 (0.69 to 2.65)	2.00 (1.11 to 3.62)	0.053	
Multivariable HR (95% CI)§¶	1 (referent)	1.35 (0.68 to 2.65)	2.01 (1.11 to 3.66)	0.045	
High (n=60)					
n	18	10	32		
Age-adjusted HR (95% CI)§	1 (referent)	1.10 (0.50 to 2.44)	2.94 (1.63 to 5.29)	< 0.001	
Multivariable HR (95% CI)§¶	1 (referent)	1.11 (0.50 to 2.44)	2.99 (1.63 to 5.49)	< 0.001	
Neoantigen loads‡					0.22
Low (n=60)					
n	22	10	28		
Age-adjusted HR (95% CI)§	1 (referent)	0.83 (0.39 to 1.78)	1.96 (1.11 to 3.45)	0.036	
Multivariable HR (95% CI)§¶	1 (referent)	0.83 (0.39 to 1.77)	1.97 (1.10 to 3.51)	0.034	
High (n=61)					
n	16	15	30		
Age-adjusted HR (95% CI)§	1 (referent)	1.76 (0.86 to 3.61)	3.08 (1.67 to 5.71)	< 0.001	
Multivariable HR (95% CI)§¶	1 (referent)	1.77 (0.87 to 3.61)	3.15 (1.68 to 5.90)	< 0.001	
Non-MSI-high					
Exome-wide tumour mutational burden‡					0.73
Low (n=292)					
n	118	96	78		
Age-adjusted HR (95% CI)§	1 (referent)	1.51 (1.15 to 1.97)	1.04 (0.78 to 1.39)	0.47	
Multivariable HR (95% CI)§¶	1 (referent)	1.56 (1.19 to 2.04)	1.00 (0.74 to 1.34)	0.83	
High (n=292)					
n	137	60	95		
Age-adjusted HR (95% CI)§	1 (referent)	0.83 (0.61 to 1.13)	1.05 (0.81 to 1.37)	0.85	
Multivariable HR (95% CI)§¶	1 (referent)	0.86 (0.63 to 1.16)	1.01 (0.77 to 1.32)	0.80	
Neoantigen loads‡					0.18
Low (n=292)					
n	117	88	87		
Age-adjusted HR (95% CI)§	1 (referent)	1.43 (1.08 to 1.88)	1.15 (0.87 to 1.52)	0.15	
Multivariable HR (95% CI)§¶	1 (referent)	1.46 (1.11 to 1.93)	1.10 (0.82 to 1.46)	0.36	
High (n=292)					
n	138	68	86		
Age-adjusted HR (95% CI)§	1 (referent)	0.93 (0.69 to 1.24)	0.97 (0.74 to 1.27)	0.62	
Multivariable HR (95% CI)§¶	1 (referent)	0.96 (0.72 to 1.29)	0.93 (0.71 to 1.23)	0.35	

* P_trend_ was calculated using a linear trend test and cumulative pack-years smoked (continuous with a ceiling at 50 pack-years).

† P_heterogeneity_ was calculated using the likelihood ratio test (1 df) for the heterogeneity of binary subtype-specific associations of cumulative pack-years smoked (continuous with a ceiling at 50 pack-years) in multivariable models.

‡ The number of MSI-high tumours containing low levels of e-TMB (or neoantigen loads) was quite small. Therefore, e-TMB was categorised into high and low based on stratum-specific median cut-off points (13 and 1.3 per megabase for MSI-high and non-MSI-high tumours, respectively). Similarly, neoantigen loads were categorised into high and low based on stratum-specific median cut-off points (2956 and 177 per exome for MSI-high and non-MSI-high tumours, respectively).

§ Inverse probability weighting was applied to reduce a potential selection bias due to the differential availability of whole-exome sequencing data (see ‘Statistical analysis’ subsection for details).

¶ The multivariable Cox regression model was adjusted for the same set of covariates as table 2.

e-TMB, exome-wide tumour mutational burden.;

## Discussion

Because colorectal carcinoma is a group of neoplasms that evolve through heterogeneous sets of genetic and epigenetic alterations influenced by lifestyle and environmental factors,^41^ the molecular pathological epidemiology (MPE) approach is useful to gain insights into the interplay of lifestyle exposures and tumour molecular alterations during the tumourigenic process.^42^,^44^ Using the PCIBM on data on long-term smoking habit and tumour WES,^20 21^ our study has shown that longitudinally updated pack-years smoked are associated with higher long-term incidence of colorectal cancer harbouring high levels of e-TMB or neoantigen loads, but not colorectal cancer containing low levels of these parameters. Such a differential association appeared to persist in MSI-high colorectal cancer. The MSI-high status has been shown to influence immune response through non-synonymous mutations and neoantigen production.^6 45 46^ Our findings suggest that tobacco compounds may make an immunosuppressive microenvironment that can favour the growth of colorectal tumours with a high burden of nonsynonymous mutations and neoantigens, providing further evidence for the suppressive effect of smoking on antitumour immunity.

The immune checkpoint inhibitors that target the CD274 (PDCD1 ligand 1, PD-L1)-PDCD1 (programmed cell death 1, PD-1) coinhibitory pathway have shown great promise in treating tumours, but their effectiveness has been confined to a limited subset of tumours including MSI-high tumours.^1^,^5^ Certain neoantigens (ie, cancer-specific antigens resulting from somatic mutations) are considered to be presented by major histocompatibility complex molecules and recognised as non-self-epitopes by T lymphocytes.^7 8^ Hence, compared with the MSI-high status, TMB and neoantigen loads are considered to be better predictors for tumour immunogenicity and responsiveness to the immune checkpoint blockade. Emerging evidence supports the predictive ability of TMB for clinical benefits from the immune checkpoint inhibitors in non-MSI-high tumours.^47^,^49^ Given the increasing availability of tumour omics profiling in clinical practice, e-TMB and neoantigen loads will likely replace tumour MSI status as a biomarker for the effectiveness of the immune checkpoint blockade.

Cigarette smoke contains thousands of DNA mutagens, which are considered to cause a distinct mutational pattern (referred to as the ‘smoking signature’) characterised by a high frequency of C–A nucleotide transversions.^17 18^ However, the smoking mutational signature commonly observed in lung carcinoma has not been well described in colorectal carcinoma. Tobacco compounds may promote carcinogenesis in various organ systems, and their mutagenic and immunosuppressive effects have been proposed as mechanisms of smoking-induced carcinogenesis.^50^,^53^ Nicotine, a major component of cigarette smoke, has been shown to impair the functions of dendritic cells and NK cells, which may promote the immune evasion of tumour cells.^51^ A previous study has shown a stronger association of smoking with colorectal cancer incidence for tumours with lower-level T cell infiltrates.^19^ Our study suggests that the immunosuppressive effect of smoking may contribute to the proliferation and survival of highly immunogenic tumour cells with abundant somatic mutations. Our analyses suggest that cumulative pack-years smoked or tumour mutational and neoantigen loads were not strongly correlated with the smoking signature in colorectal cancer. These findings further suggest that the carcinogenic effect of cigarette smoke is independent of its role in inducing mutations in the colorectal epithelium, which contrasts with the lung epithelium where smoking exerts its mutagenic effects more directly.^17 18^ Future research should examine whether prediagnosis smoking status is associated with the effectiveness of immunotherapy for colorectal cancer and whether the cessation of smoking may improve the therapeutic efficacy. Given the stronger association between cumulative pack-years smoked (or smoking cessation (inverse association)) and the incidence of colorectal cancer having higher immunogenicity, immune checkpoint inhibitors with preceding or in place of cytotoxic chemotherapy may be tested for efficacy in smokers diagnosed with TMB-high colorectal cancer. In addition, smoking cessation may be tested to determine whether it can augment the effect of immune checkpoint inhibitors for TMB-high colorectal cancer.

The current study conducted integrative MPE analyses to assess risk factor exposures and tumour molecular features.^42 43 54 55^ This MPE approach has been used to examine smoking in relation to cancer incidence/risk by tumour subtypes according to molecular pathology,^13^,^1656^ faecal microbiome^57^ and immune cell infiltrates,^19 24 58^ to shed light on the carcinogenic effects of tobacco smoke. Furthermore, the current study took advantage of the PCIBM,^20 21^ which has allowed for assessment of long-term exposures/cancer incidence by tumour subtypes.^13^,^1619 24 56 58^

Obtaining data on exome-scale TMB and neoantigen loads requires high-throughput sequencing technologies and computational analysis platforms such that no prior prospective study has examined the association of epidemiological factors with the incidence of colorectal cancer subclassified by e-TMB or neoantigen loads.^10 11^ In clinical practice, targeted sequencing assays for selected cancer-associated gene panels are increasingly used, and the term ‘TMB’ is commonly used for a mutational frequency in the selected genes. However, pathogenic mutations in those selected cancer-associated genes are subject to substantial selection pressure, which causes imprecision when estimating the e-TMB, neoantigen load and tumour immunogenicity.^10 11^ In addition, gene panels in targeted sequencing assays documented in the literature differ from study to study, which makes cross-comparisons challenging. To avoid these problems, we used WES to capture exome-wide mutational profile and better estimate the tumour immunogenicity. Because of the increasing availability and reducing costs of omics assays, personalised treatment of cancer patients based on comprehensive exomic mutational profiling will likely be implemented in the future. In the I-PREDICT studies, patients with treatment-naïve or refractory malignancy were treated with agents matched for identified molecular alterations, and targeting a larger fraction of the alterations resulted in better survival outcomes.^59 60^ In parallel with the trend of precision oncology, our study highlights the potential of the exome-wide assessment of tumour immunogenicity in epidemiological research.

The current study has limitations. First, there is the possibility of unmeasured and/or residual confounding. Nonetheless, we adjusted for many established risk factors of colorectal cancer in our multivariable models, and this adjustment did not alter our findings materially from those of the univariable analyses. It is also noted that randomising smoking exposure in a trial study to eliminate confounding would be unethical and thus impossible. Second, the neoantigen loads were estimated using in silico methods that depended on HLA class I predictions. Using the method, a previous study found that the neoantigen load from WES data robustly correlated with lymphocytic reaction levels in colorectal cancer.^6^ Nonetheless, the data on neoantigen load had certain measurement errors and should be replicated in independent studies. Moreover, we could examine e-TMB as another measure of tumour immunogenicity, which yielded similar data to those using the neoantigen loads. Third, WES data were not available for many colorectal cancer cases within the cohorts, which might have caused selection bias. However, we used all of the 3053 incident colorectal cancers and the IPW method to adjust for the selection bias,^40^ and analyses with and without the IPW adjustment yielded similar results. Fourth, most study participants were Caucasian health professionals, and therefore, our findings need to be validated in independent populations.

The current study has notable strengths. First, our prospective cohort design enabled us to obtain longitudinally updated data on smoking habits and potential confounders, while eliminating differential recall bias between cancer cases and cancer-free individuals. Second, the prospective study design also enabled us to use the 3053 incident colorectal cancers to adjust for selection bias due to the availability of the WES data. Third, in contrast to commonly used targeted sequencing assays, WES analyses could yield exome-scale TMB and neoantigen loads. Fourth, integrated MPE analyses using the PCIBM on the prospective cohort studies with the WES data in incident tumours could assess not only the longitudinal effect of smoking on the long-term incidence of WES-based tumour subtypes but also statistical heterogeneity between the subtypes. Fifth, our database also enabled us to evaluate results after controlling for other key tumour characteristics such as MSI status and T cell infiltrates. Sixth, our incident colorectal cancer cases represented a collection of patients who visited hundreds of different hospitals throughout the USA, which provided higher generalisability compared with studies based on only a limited number of hospitals.

## Conclusions

The current study demonstrated a stronger association of smoking with colorectal cancer incidence for tumours containing higher numbers of exome-wide somatic mutations. Smoking may contribute to the development of colorectal tumours, especially those with high frequencies of somatic mutations, possibly through its effect on the tumour immune microenvironment. Future studies should examine whether cessation of smoking may stimulate antitumour immune response and improve response to immune checkpoint inhibitors. As omics analysis platforms are increasingly available and cost-efficient for routine tumour pathological testing, the current study will inform the effort of implementing tumour exome sequencing analyses in epidemiological research and clinical practice.

## Supplementary material

10.1136/bmjonc-2025-000787 online supplemental file 1

## Data Availability

Data are available on reasonable request.
