# Supplementary material for: Smoking habit and long-term colorectal cancer incidence by exome-wide mutational and neoantigen loads: evidence based on the prospective cohort incident-tumour biobank method
Source: BMJ Oncol. 2025 Jun 3;4(1):e000787. doi: 10.1136/bmjonc-2025-000787 (PMC12164326; doi:10.1136/bmjonc-2025-000787)
Supplement: online supplemental file 1 [file bmjonc-4-1-s001.docx]

**
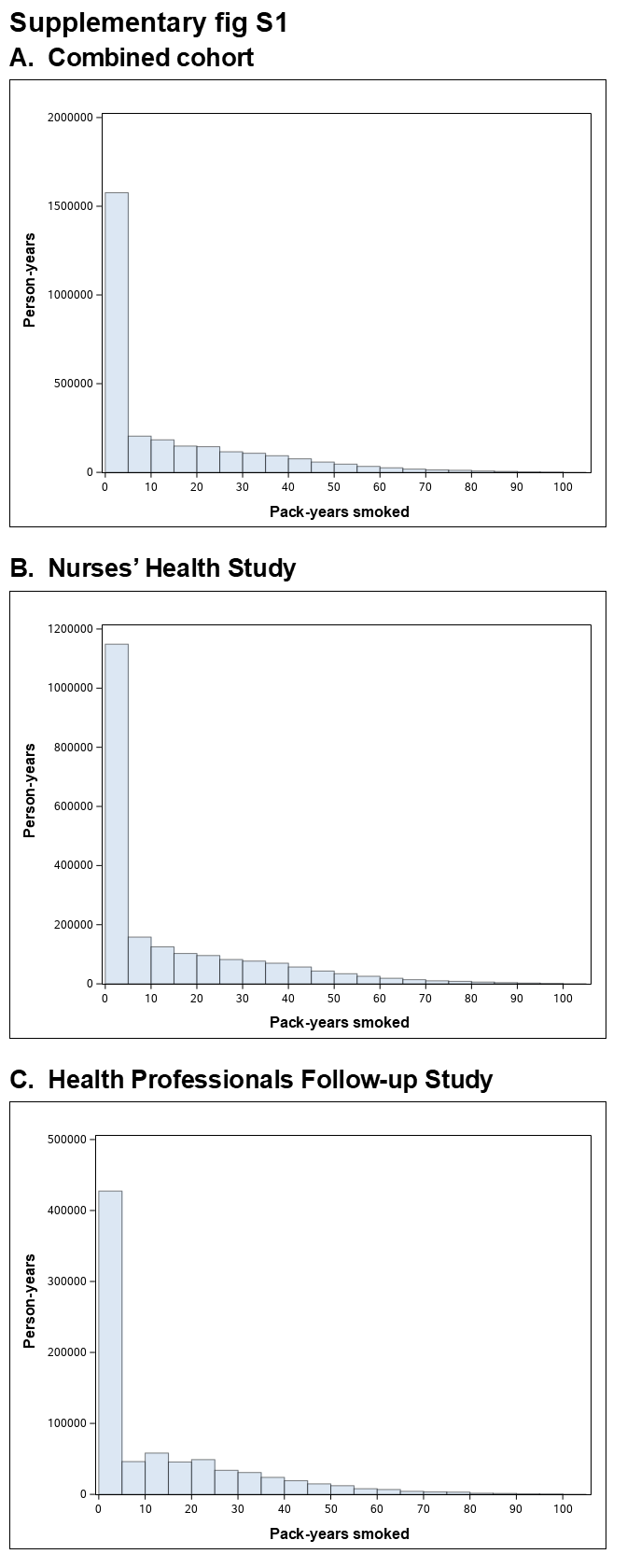
**

Supplementary fig S1. Histograms of cumulative pack-years smoked among participants in the Nurses’ Health Study and the Health Professionals Follow-up Study. **A.** Combined cohort. **B.** The Nurses’ Health Study (women). **C.** The Health Professionals Follow-up Study (men).


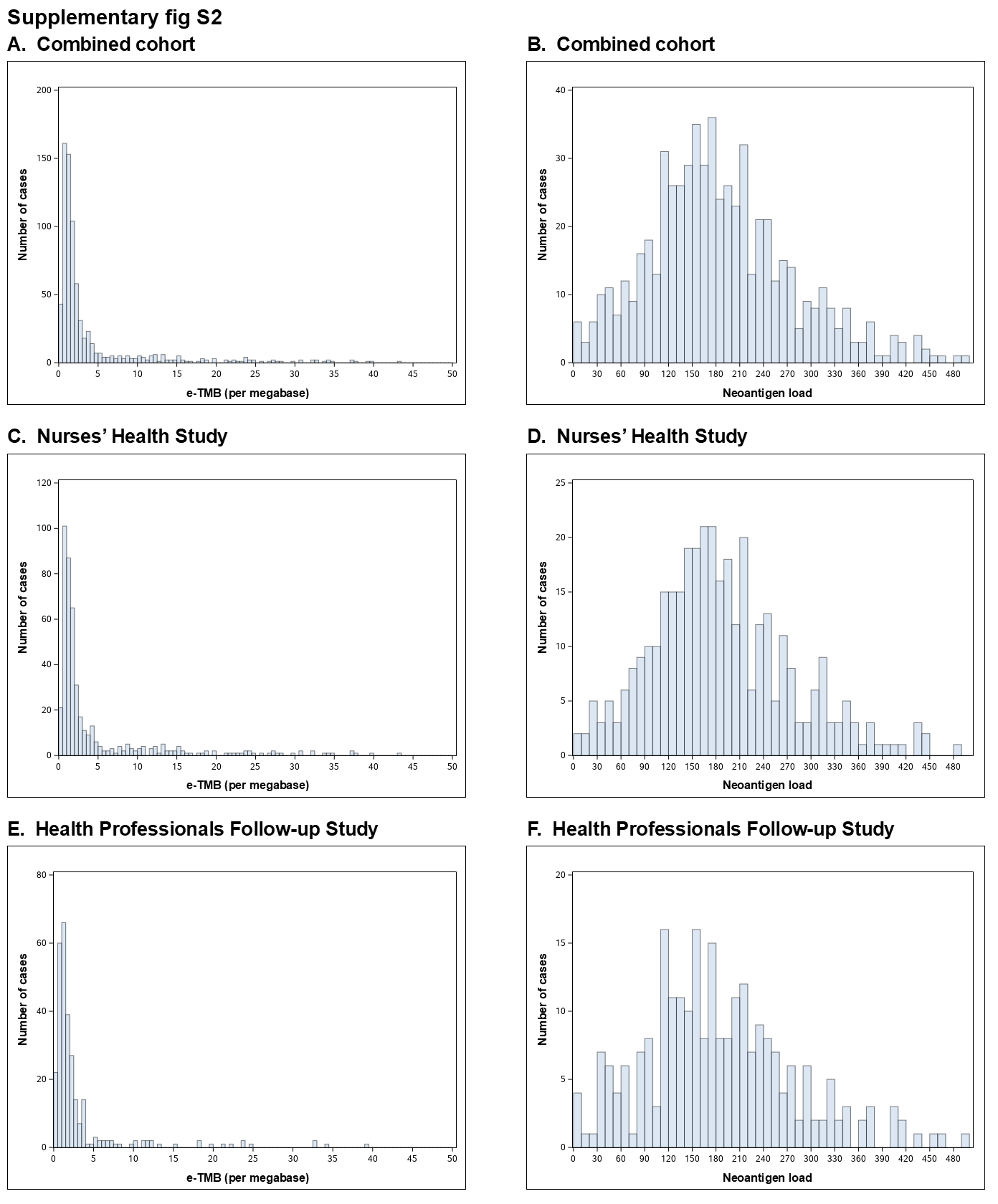


Supplementary fig S2. Histograms of exome-wide tumour mutational burden (e-TMB) and neoantigen loads. **A.** e-TMB in the combined cohort. **B.** Neoantigen loads in the combined cohort. **C.** e-TMB in the Nurses’ Health Study.  **D.** Neoantigen loads in the Nurses’ Health Study. **E.** e-TMB in the Health Professionals Follow-up Study. **F.** Neoantigen loads in the Health Professionals Follow-up Study.

e-TMB, exome-wide tumour mutational burden.


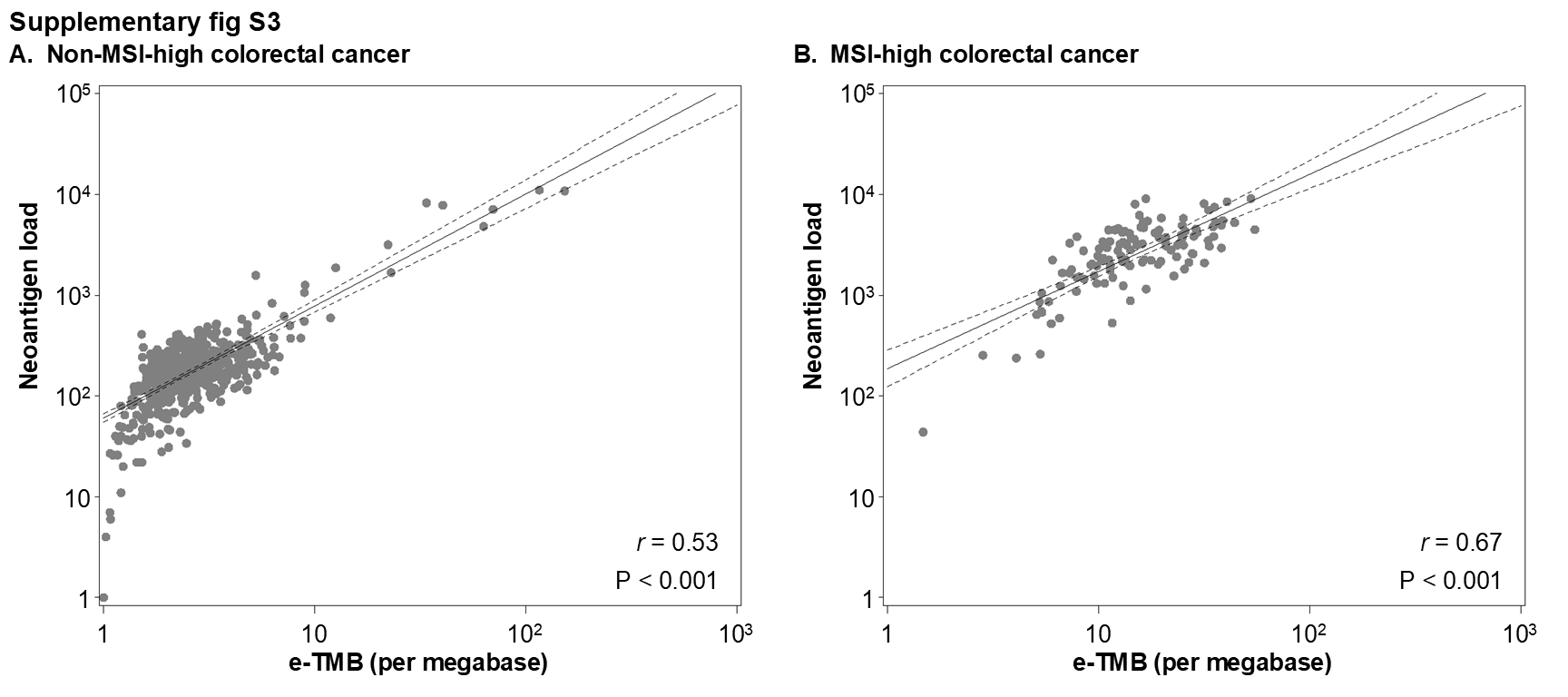


Supplementary fig S3. Correlation plots of exome-wide tumour mutational burden and neoantigen loads by tumour status of microsatellite instability. The correlation coefficient (*r*) and *P* value were calculated using the Spearman correlation test. Dotted lines indicate 95% confidence interval of the regression line.

e-TMB, exome-wide tumour mutational burden; MSI, microsatellite instability.

**
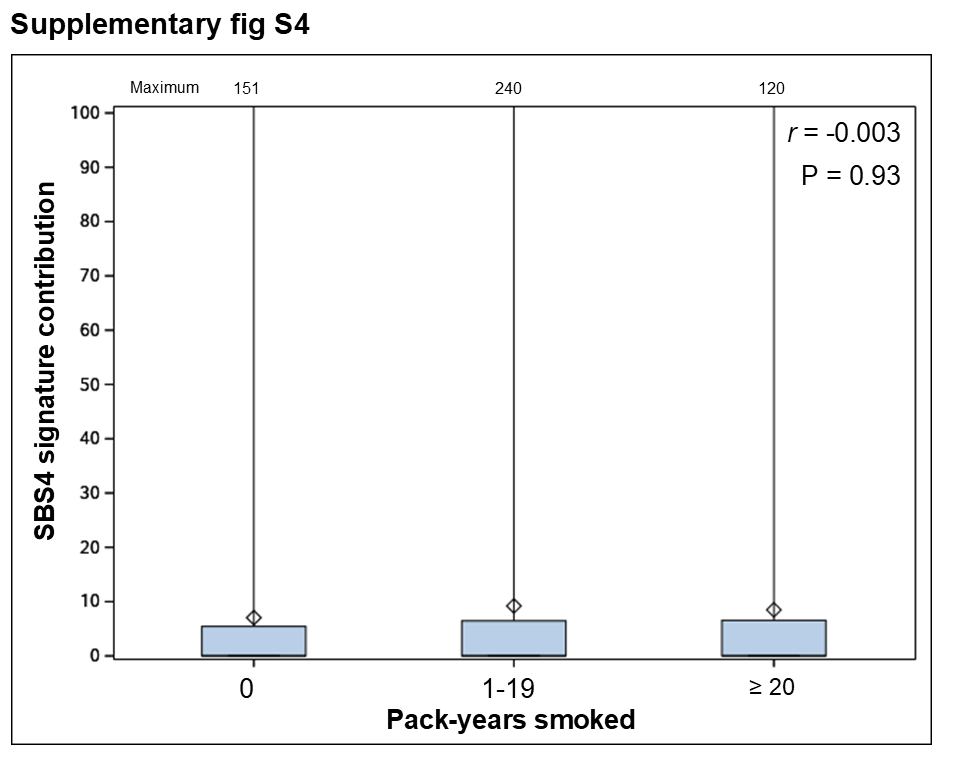
**

Supplementary fig S4. Box plots of smoking signature (SBS4 signature contribution) according to cumulative pack-years smoked. The correlation coefficient (*r*) and *P* value was calculated using the Spearman correlation test.

e-TMB, exome-wide tumour mutational burden; SBS, single base substitution.


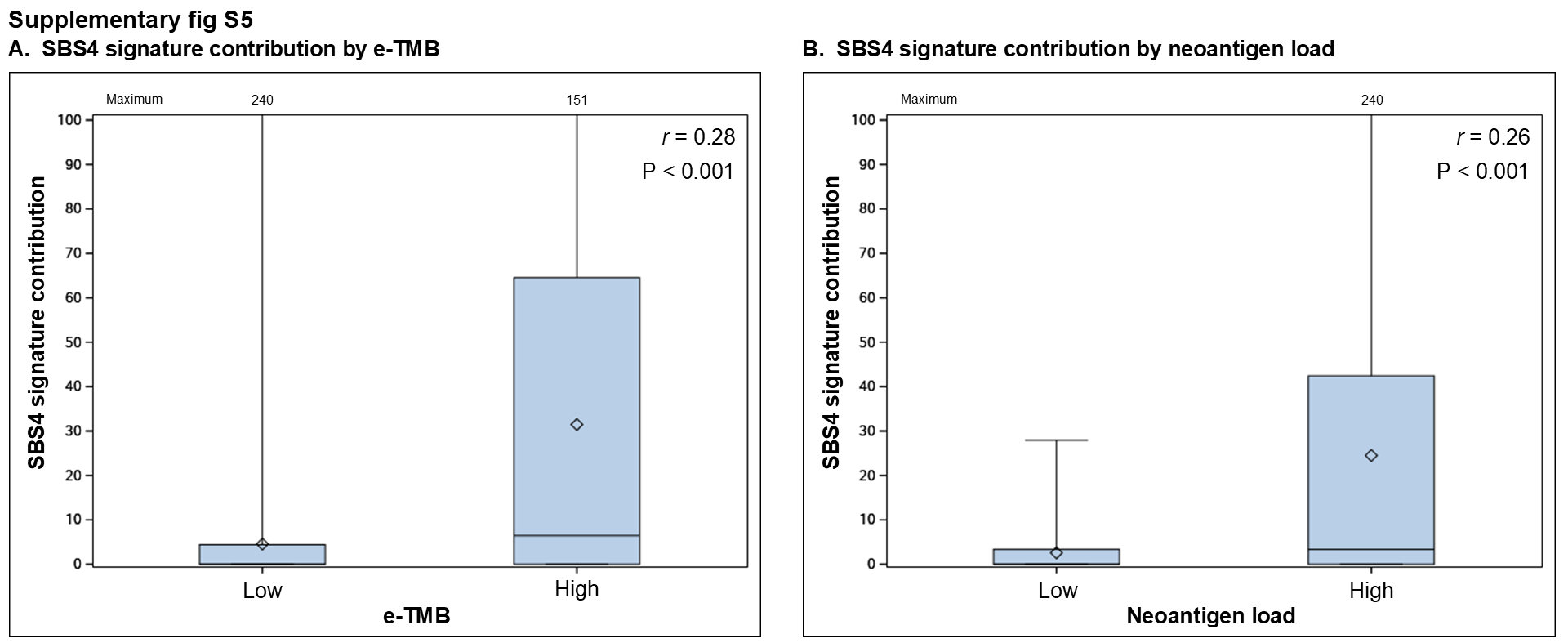


Supplementary fig S5. Box plots of smoking signature (SBS4 signature contribution) according to exome-wide tumour mutational burden (e-TMB) and neoantigen loads. **A.** By e-TMB. **B.** By neoantigen loads. The correlation coefficient (*r*) and *P* value was calculated using the Spearman correlation test.

e-TMB, exome-wide tumour mutational burden; SBS, single base substitution.


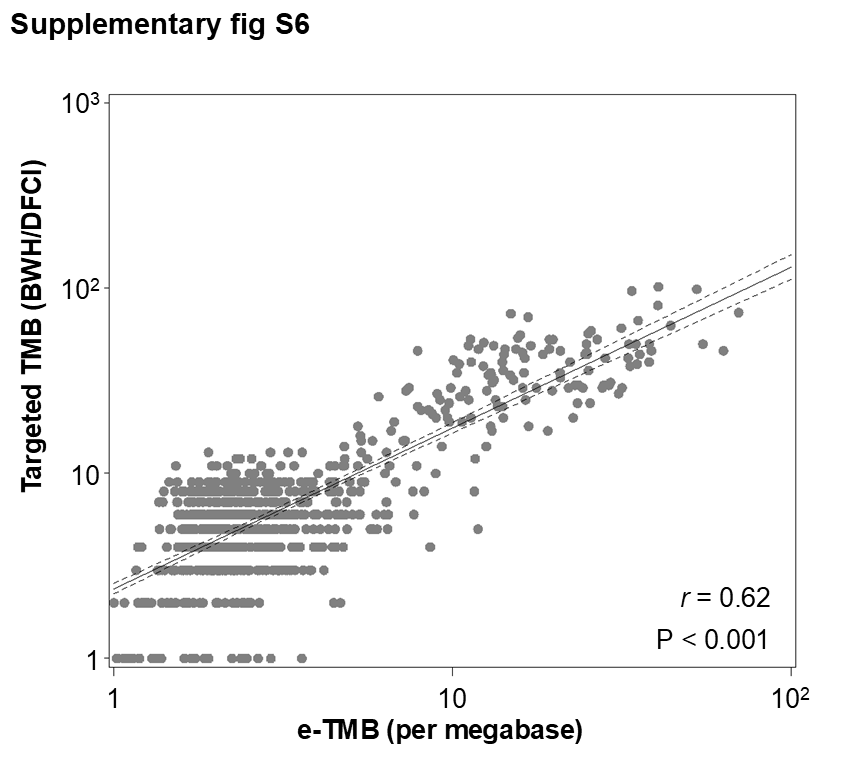


Supplementary fig S6. Correlation plot of exome-wide tumour mutational burden and targeted tumour mutational burden based on a clinical panel of 447 selected cancer-associated genes used in the Brigham and Women’s Hospital. The correlation coefficient (*r*) and *P* value were calculated using the Spearman correlation test. Dotted lines indicate 95% confidence interval of the regression line.

BWH, the Brigham and Women’s Hospital; DFCI, Dana-Farber Cancer Institute; e-TMB, exome-wide tumour mutational burden.

Supplementary table S1. Age-standardised characteristics of colorectal cancer cases according to availability of whole-exome sequencing data in the Nurses’ Health Study and Health Professionals Follow-up Study

|  | NHS | |  | HPFS | |
| --- | --- | --- | --- | --- | --- |
|  | WES data | |  | WES data | |
| Characteristic* | Available | Unavailable |  | Available | Unavailable |
| n | 462 | 1,306 |  | 290 | 995 |
| Age, years | 68.1 (8.4) | 65.4 (9.6) |  | 70.9 (9.2) | 69.9 (9.5) |
| Family history of colorectal cancer | 21% | 20% |  | 20% | 17% |
| History of diabetes | 9.6% | 11% |  | 11% | 12% |
| Body mass index, kg/m² | 26.0 (4.8) | 26.1 (5.0) |  | 26.1 (3.4) | 26.3 (3.2) |
| Postmenopause | 92% | 92% |  | - | - |
| Menopausal hormone therapy | 26% | 18% |  | - | - |
| History of colonoscopy/sigmoidoscopy | 42% | 34% |  | 46% | 46% |
| Regular use of multivitamins | 57% | 49% |  | 47% | 45% |
| Regular use of aspirin | 40% | 39% |  | 45% | 45% |
| Regular use of other NSAIDs | 22% | 15% |  | 14% | 13% |
| Physical activity, METS-hours/week | 15.9 (17.2) | 15.5 (16.3) |  | 24.4 (19.0) | 24.2 (22.1) |
| Cumulative pack-years smoked^†^ |  |  |  |  |  |
| 0 | 40% | 43% |  | 46% | 40% |
| 1-19 | 25% | 24% |  | 24% | 23% |
| ≥ 20 | 35% | 33% |  | 30% | 37% |
| Total calorie intake, kcal/day | 1,715 (434) | 1,671 (437) |  | 1,961 (494) | 1,974 (574) |
| Alcohol intake, g/day | 5.4 (8.0) | 6.8 (10.5) |  | 13.3 (15.8) | 13.3 (15.9) |
| Red and processed meat intake, servings/week | 6.1 (2.9) | 6.5 (3.8) |  | 6.6 (3.8) | 6.6 (4.4) |
| Total calcium intake, mg/day | 940 (343) | 907 (335) |  | 925 (384) | 928 (393) |
| Total folate intake, μg/day | 425 (158) | 415 (192) |  | 517 (222) | 534 (240) |
| Alternate Healthy Eating Index 2010^‡^ | 46.6 (8.6) | 46.9 (9.3) |  | 48.1 (10.9) | 48.0 (9.9) |
| Tumour location^†^ |  |  |  |  |  |
| Proximal colon | 51% | 46% |  | 48% | 39% |
| Distal colon | 29% | 30% |  | 31% | 36% |
| Rectum | 20% | 24% |  | 21% | 26% |
| AJCC disease stage^†^ |  |  |  |  |  |
| I | 26% | 24% |  | 27% | 30% |
| II | 35% | 32% |  | 26% | 29% |
| III | 28% | 27% |  | 31% | 26% |
| IV | 11% | 17% |  | 15% | 15% |

* All variables other than age were standardised to age distribution of each cohort. Mean (standard deviation) was presented for continuous variables.

^†^ Total percentages may not equal 100% due to rounding.

^‡^ Without alcohol intake.

AJCC, American Joint Committee on Cancer; HPFS, Health Professionals Follow-up Study; METS, metabolic equivalent task score; NHS, Nurses’ Health Study; NSAID, nonsteroidal anti-inflammatory drug; WES, whole-exome sequencing.

Supplementary table S2. Selected cancer-associated genes used to calculate tumour mutational burden (“targeted TMB”) in the current study

| *ABCB11* | *ABL1* | *ACVR1* | *AKT1* | *AKT2* | *AKT3* | *ALK* | *APC* |
| --- | --- | --- | --- | --- | --- | --- | --- |
| *AR* | *ARAF* | *ARHGAP35* | *ARHGEF12* | *ARID1A* | *ARID1B* | *ARID2* | *ASXL1* |
| *ATM* | *ATR* | *ATRX* | *AURKA* | *AURKB* | *AXIN2* | *AXL* | *B2M* |
| *BABAM1* | *BAP1* | *BARD1* | *BCL11B* | *BCL2* | *BCL2L1* | *BCL2L12* | *BCL6* |
| *BCOR* | *BCORL1* | *BLM* | *BMPR1A* | *BRAF* | *BRCA1* | *BRCA2* | *BRCC3* |
| *BRD3* | *BRD4* | *BRE* | *BRIP1* | *BUB1B* | *C17ORF70* | *C19ORF40* | *C1ORF86* |
| *CALR* | *CARD11* | *CASP8* | *CBFA2T3* | *CBFB* | *CBL* | *CBLB* | *CCND1* |
| *CCND2* | *CCND3* | *CCNE1* | *CD274* | *CD79B* | *CDC73* | *CDH1* | *CDH4* |
| *CDK12* | *CDK4* | *CDK6* | *CDK8* | *CDKN1A* | *CDKN1B* | *CDKN1C* | *CDKN2A* |
| *CDKN2B* | *CDKN2C* | *CEBPA* | *CHEK1* | *CHEK2* | *CIC* | *CIITA* | *COL7A1* |
| *CREBBP* | *CRKL* | *CRLF2* | *CRTC1* | *CSF3R* | *CTCF* | *CTLA4* | *CTNNA1* |
| *CTNNB1* | *CUX1* | *CXCR4* | *CYLD* | *DAXX* | *DCLRE1C* | *DDB1* | *DDB2* |
| *DDR2* | *DICER1* | *DIS3* | *DIS3L2* | *DKC1* | *DMC1* | *DNMT3A* | *DOCK8* |
| *EGFR* | *EGLN1* | *ELANE* | *EME1* | *ENG* | *EP300* | *EPCAM* | *ERBB2* |
| *ERBB3* | *ERBB4* | *ERCC1* | *ERCC2* | *ERCC3* | *ERCC4* | *ERCC5* | *ERCC6* |
| *ERG* | *ESR1* | *ETV1* | *ETV4* | *ETV5* | *ETV6* | *EWSR1* | *EXO1* |
| *EXT1* | *EXT2* | *EZH2* | *FAH* | *FAM175A* | *FAM46C* | *FAN1* | *FANCA* |
| *FANCB* | *FANCC* | *FANCD2* | *FANCE* | *FANCF* | *FANCG* | *FANCI* | *FANCL* |
| *FANCM* | *FAS* | *FAT1* | *FBXW7* | *FGFR1* | *FGFR2* | *FGFR3* | *FGFR4* |
| *FH* | *FLCN* | *FLT1* | *FLT3* | *FLT4* | *FOXA1* | *FOXL2* | *FUS* |
| *GALNT12* | *GATA2* | *GATA3* | *GATA4* | *GATA6* | *GBA* | *GEN1* | *GLI1* |
| *GLI2* | *GNA11* | *GNAQ* | *GNAS* | *GPC3* | *GREM1* | *H19* | *H3F3A* |
| *H3F3B* | *HABP2* | *HELQ* | *HFE* | *HIST1H3B* | *HIST1H3C* | *HMBS* | *HNF1A* |
| *HOXB13* | *HRAS* | *ID3* | *ID4* | *IDH1* | *IDH2* | *IGF1R* | *IGF2* |
| *IKZF1* | *IL7R* | *ITK* | *JAK1* | *JAK2* | *JAK3* | *JAZF1* | *KAT6A* |
| *KAT6B* | *KCNQ1* | *KDM5A* | *KDM5C* | *KDM6A* | *KDR* | *KEAP1* | *KIF1B* |
| *KIT* | *KLF2* | *KLF4* | *KLLN* | *KMT2A* | *KMT2D* | *KRAS* | *LIG4* |
| *LMO1* | *LMO2* | *MAF* | *MAFB* | *MAP2K1* | *MAP2K2* | *MAP2K4* | *MAP3K1* |
| *MAPK1* | *MAX* | *MBD4* | *MCL1* | *MCM8* | *MDM2* | *MDM4* | *MECOM* |
| *MED12* | *MEF2B* | *MEN1* | *MET* | *MGA* | *MITF* | *MLH1* | *MLH3* |
| *MPL* | *MRE11A* | *MSH2* | *MSH6* | *MTA1* | *MTAP* | *MTOR* | *MUS81* |
| *MUTYH* | *MYB* | *MYBL1* | *MYC* | *MYCL1* | *MYCN* | *MYD88* | *NBN* |
| *NEIL1* | *NEIL2* | *NEIL3* | *NF1* | *NF2* | *NFE2L2* | *NFKBIA* | *NFKBIE* |
| *NFKBIZ* | *NKX2-1* | *NKX3-1* | *NOTCH1* | *NOTCH2* | *NOTCH3* | *NPM1* | *NR0B1* |
| *NRAS* | *NRG1* | *NSD1* | *NT5C2* | *NTHL1* | *NTRK1* | *NTRK2* | *NTRK3* |
| *OGG1* | *PALB2* | *PARK2* | *PAX5* | *PAXIP1* | *PBRM1* | *PDCD1LG2* | *PDGFRA* |
| *PDGFRB* | *PHF6* | *PHOX2B* | *PIK3C2B* | *PIK3CA* | *PIK3R1* | *PIM1* | *PML* |
| *PMS1* | *PMS2* | *PNKP* | *POLB* | *POLD1* | *POLE* | *POLH* | *POLQ* |
| *POT1* | *PPARG* | *PPM1D* | *PPP2R1A* | *PRDM1* | *PRF1* | *PRKAR1A* | *PRKCI* |
| *PRKDC* | *PRSS1* | *PTCH1* | *PTEN* | *PTK2B* | *PTPN11* | *PTPN14* | *PVRL4* |
| *QKI* | *RAC1* | *RAD21* | *RAD50* | *RAD51* | *RAD51C* | *RAD51D* | *RAD52* |
| *RAD54B* | *RAF1* | *RARA* | *RASA1* | *RB1* | *RBBP8* | *RBM10* | *RECQL4* |
| *REL* | *RELA* | *RET* | *RHBDF2* | *RHEB* | *RHOA* | *RHOH* | *RHOT1* |
| *RICTOR* | *RIF1* | *RINT1* | *RIT1* | *RMRP* | *RNF43* | *RNF8* | *ROS1* |
| *RPA1* | *RPTOR* | *RSPO2* | *RSPO3* | *RUNX1* | *RUNX1T1* | *SBDS* | *SDHA* |
| *SDHAF2* | *SDHB* | *SDHC* | *SDHD* | *SERPINA1* | *SETBP1* | *SETD2* | *SF3B1* |
| *SH2B3* | *SH2D1A* | *SLC25A13* | *SLC34A2* | *SLX1A* | *SLX1B* | *SLX4* | *SMAD2* |
| *SMAD4* | *SMARCA4* | *SMARCB1* | *SMARCE1* | *SMC3* | *SMO* | *SOCS1* | *SOS1* |
| *SOX2* | *SOX9* | *SPOP* | *SRSF2* | *SRY* | *SS18* | *STAG2* | *STAT3* |
| *STAT6* | *STK11* | *SUFU* | *SUZ12* | *TAL1* | *TAL2* | *TAZ* | *TCEB1* |
| *TCF3* | *TCF7L2* | *TDG* | *TERC* | *TERT* | *TET1* | *TET2* | *TFE3* |
| *TLX3* | *TMEM127* | *TMPRSS2* | *TNFAIP3* | *TOPBP1* | *TP53* | *TP53BP1* | *TRAF3* |
| *TRAF7* | *TRIM37* | *TSC1* | *TSC2* | *TSHR* | *U2AF1* | *UBE2T* | *UIMC1* |
| *UROD* | *USP28* | *USP8* | *VEGFA* | *VHL* | *WAS* | *WHSC1* | *WHSC1L1* |
| *WRN* | *WT1* | *XPA* | *XPC* | *XPO1* | *XRCC1* | *XRCC2* | *XRCC3* |
| *XRCC4* | *XRCC5* | *XRCC6* | *YAP1* | *ZNF217* | *ZNRF3* | *ZRSR2* |  |

Supplementary table S3. Cumulative pack-years smoked and colorectal cancer incidence in the Nurses’ Health Study and the Health Professionals Follow-up Study, overall and by availability of whole-exome sequencing data

|  | Cumulative pack-years smoked | | |  |
| --- | --- | --- | --- | --- |
|  | 0 | 1-19 | ≥ 20 | P_trend_^†^ |
| Person-years | 1,609,012 | 936,934 | 927,496 |  |
|  |  |  |  |  |
| **All colorectal cancer** (n *=* 3,053) |  |  |  |  |
| n | 1,277 | 732 | 1,044 |  |
| Age-adjusted HR (95% CI) | 1 (referent) | 1.08 (0.98-1.18) | 1.24 (1.14-1.35) | < 0.001 |
| Multivariable HR (95% CI)* | 1 (referent) | 1.08 (0.98-1.18) | 1.16 (1.07-1.27) | < 0.001 |
|  |  |  |  |  |
| **Colorectal cancer with WES data** (n *=* 752) |  |  |  |  |
| n | 316 | 189 | 247 |  |
| Age-adjusted HR (95% CI) | 1 (referent) | 1.00 (0.83-1.21) | 1.25 (1.06-1.47) | < 0.001 |
| Multivariable HR (95% CI)* | 1 (referent) | 0.99 (0.82-1.19) | 1.16 (0.98-1.37) | 0.022 |
|  |  |  |  |  |
| **Colorectal cancer without WES data** (n *=* 2,301) |  |  |  |  |
| n | 961 | 543 | 797 |  |
| Age-adjusted HR (95% CI) | 1 (referent) | 1.12 (0.94-1.35) | 1.20 (1.02-1.43) | 0.012 |
| Multivariable HR (95% CI)* | 1 (referent) | 1.12 (0.93-1.34) | 1.12 (0.94-1.32) | 0.15 |
|  |  |  |  |  |

* The multivariable Cox regression model was adjusted for the same set of covariates as Table 2.

^†^ P_trend_ was calculated using a linear trend test and cumulative pack-years smoked (continuous with a ceiling at 50 pack-years).

CI, confidence interval; HR, hazard ratio; WES, whole-exome sequencing.

Supplementary table S4. Cumulative pack-years smoked and colorectal cancer incidence, overall and by exome-wide tumour mutational burden or neoantigen loads, in the Nurses’ Health Study and the Health Professionals Follow-up Study

|  | Cumulative pack-years smoked | | |  |  |
| --- | --- | --- | --- | --- | --- |
|  | 0 | 1-19 | ≥ 20 | P_trend_^§^ | P_heterogeneity_^¶^ |
| **Nurses’ Health Study** |  |  |  |  |  |
|  |  |  |  |  |  |
| Person-years | 1,125,146 | 705,774 | 661,296 |  |  |
|  |  |  |  |  |  |
| **Exome-wide tumour mutational burden*** | |  |  |  | 0.004 |
| Low (n = 389) |  |  |  |  |  |
| n | 161 | 105 | 123 |  |  |
| Age-adjusted HR (95% CI)^†^ | 1 (referent) | 1.19 (0.93-1.53) | 1.29 (1.02-1.64) | 0.062 |  |
| Multivariable HR (95% CI)^†‡^ | 1 (referent) | 1.26 (0.98-1.62) | 1.32 (1.04-1.69) | 0.065 |  |
| High (n = 73) |  |  |  |  |  |
| n | 22 | 14 | 37 |  |  |
| Age-adjusted HR (95% CI)^†^ | 1 (referent) | 1.15 (0.58-2.30) | 2.79 (1.64-4.77) | < 0.001 |  |
| Multivariable HR (95% CI)^†‡^ | 1 (referent) | 1.22 (0.61-2.42) | 2.88 (1.68-4.93) | < 0.001 |  |
|  |  |  |  |  |  |
| **Neoantigen loads*** |  |  |  |  | 0.020 |
| Low (n = 346) |  |  |  |  |  |
| n | 143 | 97 | 106 |  |  |
| Age-adjusted HR (95% CI)^†^ | 1 (referent) | 1.24 (0.96-1.61) | 1.25 (0.97-1.61) | 0.094 |  |
| Multivariable HR (95% CI)^†‡^ | 1 (referent) | 1.31 (1.01-1.70) | 1.28 (0.99-1.66) | 0.094 |  |
| High (n = 116) |  |  |  |  |  |
| n | 40 | 22 | 54 |  |  |
| Age-adjusted HR (95% CI)^†^ | 1 (referent) | 1.00 (0.59-1.70) | 2.28 (1.51-3.44) | < 0.001 |  |
| Multivariable HR (95% CI)^†‡^ | 1 (referent) | 1.05 (0.62-1.79) | 2.34 (1.54-3.55) | < 0.001 |  |
|  |  |  |  |  |  |
| **Health Professionals Follow-up Study** | |  |  |  |  |
|  |  |  |  |  |  |
| Person-years | 483,866 | 231,160 | 266,200 |  |  |
|  |  |  |  |  |  |
| **Exome-wide tumour mutational burden*** | |  |  |  | 0.062 |
| Low (n = 265) |  |  |  |  |  |
| n | 125 | 64 | 76 |  |  |
| Age-adjusted HR (95% CI)^†^ | 1 (referent) | 1.00 (0.74-1.35) | 0.80 (0.60-1.07) | 0.31 |  |
| Multivariable HR (95% CI)^†‡^ | 1 (referent) | 0.99 (0.73-1.33) | 0.72 (0.53-0.96) | 0.061 |  |
| High (n = 25) |  |  |  |  |  |
| n | 8 | 6 | 11 |  |  |
| Age-adjusted HR (95% CI)^†^ | 1 (referent) | 1.56 (0.54-4.55) | 2.02 (0.80-5.09) | 0.099 |  |
| Multivariable HR (95% CI)^†‡^ | 1 (referent) | 1.58 (0.55-4.52) | 1.81 (0.72-4.55) | 0.18 |  |
|  |  |  |  |  |  |
|  |  |  |  |  |  |
|  |  |  |  |  |  |
|  |  |  |  |  |  |
|  |  |  |  |  |  |
|  |  |  |  |  |  |
|  |  |  |  |  |  |
|  |  |  |  |  |  |
| **Neoantigen loads*** |  |  |  |  | 0.31 |
| Low (n = 217) |  |  |  |  |  |
| n | 101 | 54 | 62 |  |  |
| Age-adjusted HR (95% CI)^†^ | 1 (referent) | 1.02 (0.73-1.42) | 0.80 (0.58-1.10) | 0.39 |  |
| Multivariable HR (95% CI)^†‡^ | 1 (referent) | 1.00 (0.72-1.39) | 0.71 (0.51-0.99) | 0.091 |  |
| High (n = 73) |  |  |  |  |  |
| n | 32 | 16 | 25 |  |  |
| Age-adjusted HR (95% CI)^†^ | 1 (referent) | 1.07 (0.58-1.95) | 1.09 (0.65-1.84) | 0.62 |  |
| Multivariable HR (95% CI)^†‡^ | 1 (referent) | 1.09 (0.60-1.99) | 1.02 (0.61-1.71) | 0.89 |  |
|  |  |  |  |  |  |

* e-TMB was categorised into high (≥ 10 per megabase) and low (< 10 per megabase). Based on all colorectal cancer cases with available whole-exome sequencing data in each cohort, neoantigen loads were categorised into high (≥ 375 and ≥ 291 per exome for the NHS and HPFS, respectively; the top quartile) and low (< 375 and < 291 per exome for the NHS and HPFS, respectively; the other quartiles).

^†^ Inverse probability weighting was applied to reduce a potential selection bias due to the differential availability of whole-exome sequencing data (see “Statistical analysis” subsection for details).

^‡^ The multivariable Cox regression model was adjusted for the same set of covariates as Table 2.

^§^ P_trend_ was calculated using a linear trend test and cumulative pack-years smoked (continuous with a ceiling at 50 pack-years).

^¶^ P_heterogeneity_ was calculated using the likelihood ratio test (one degree of freedom) for the heterogeneity of binary subtype-specific associations of cumulative pack-years smoked (continuous with a ceiling at 50 pack-years) in multivariable models.

CI, confidence interval; HPFS, Health Professionals Follow-up Study; HR, hazard ratio; NHS, Nurses’ Health Study.

Supplementary table S5. Cumulative pack-years smoked and colorectal cancer incidence by exome-wide tumour mutational burden or neoantigen loads, without inverse probability weighting (IPW)

|  | Cumulative pack-years smoked | | |  |  |
| --- | --- | --- | --- | --- | --- |
|  | 0 | 1-19 | ≥ 20 | P_trend_^‡^ | P_heterogeneity_^§^ |
| **Exome-wide tumour mutational burden*** | |  |  |  | < 0.001 |
| Low (n = 654) |  |  |  |  |  |
| n | 286 | 169 | 199 |  |  |
| Age-adjusted HR (95% CI) | 1 (referent) | 1.11 (0.92-1.34) | 1.07 (0.89-1.28) | 0.33 |  |
| Multivariable HR (95% CI)^†^ | 1 (referent) | 1.13 (0.93-1.37) | 1.02 (0.84-1.23) | 0.76 |  |
| High (n = 98) |  |  |  |  |  |
| n | 30 | 20 | 48 |  |  |
| Age-adjusted HR (95% CI) | 1 (referent) | 1.24 (0.70-2.19) | 2.55 (1.61-4.04) | < 0.001 |  |
| Multivariable HR (95% CI)^†^ | 1 (referent) | 1.27 (0.72-2.24) | 2.47 (1.56-3.92) | < 0.001 |  |
|  |  |  |  |  |  |
| **Neoantigen loads*** |  |  |  |  | 0.027 |
| Low (n = 564) |  |  |  |  |  |
| n | 242 | 154 | 168 |  |  |
| Age-adjusted HR (95% CI) | 1 (referent) | 1.18 (0.96-1.44) | 1.06 (0.86-1.29) | 0.31 |  |
| Multivariable HR (95% CI)^†^ | 1 (referent) | 1.20 (0.97-1.47) | 1.01 (0.82-1.23) | 0.70 |  |
| High (n = 188) |  |  |  |  |  |
| n | 74 | 35 | 79 |  |  |
| Age-adjusted HR (95% CI) | 1 (referent) | 0.92 (0.61-1.38) | 1.71 (1.24-2.36) | 0.001 |  |
| Multivariable HR (95% CI)^†^ | 1 (referent) | 0.95 (0.64-1.43) | 1.66 (1.20-2.30) | 0.004 |  |
|  |  |  |  |  |  |

* e-TMB was categorised into high (≥ 10 per megabase) and low (< 10 per megabase). Based on all colorectal cancer cases with available whole-exome sequencing data, neoantigen loads were categorised into high (≥ 326 per exome, the top quartile) and low (< 326 per exome, the other quartiles).

^†^ The multivariable Cox regression model was adjusted for the same set of covariates as Table 2.

^‡^ P_trend_ was calculated using a linear trend test and cumulative pack-years smoked (continuous with a ceiling at 50 pack-years).

^§^ P_heterogeneity_ was calculated using the likelihood ratio test (one degree of freedom) for the heterogeneity of binary subtype-specific associations of cumulative pack-years smoked (continuous with a ceiling at 50 pack-years) in multivariable models.

CI, confidence interval; e-TMB, exome-wide tumour mutational burden; HR, hazard ratio.

Supplementary table S6. Cumulative pack-years smoked and colorectal cancer incidence by tumour mutational burden based on selected cancer-associated genes [“targeted tumour mutational burden (TMB)”]

|  | Cumulative pack-years smoked | | |  |  |
| --- | --- | --- | --- | --- | --- |
|  | 0 | 1-19 | ≥ 20 | P_trend_^§^ | P_heterogeneity_^¶^ |
| **Targeted TMB (BWH / DFCI)*** | |  |  |  | 0.034 |
| Low (n = 541) |  |  |  |  |  |
| n | 234 | 143 | 164 |  |  |
| Age-adjusted HR (95% CI)^†^ | 1 (referent) | 1.16 (0.94-1.42) | 1.08 (0.88-1.32) | 0.35 |  |
| Multivariable HR (95% CI)^†‡^ | 1 (referent) | 1.19 (0.96-1.47) | 1.05 (0.85-1.30) | 0.60 |  |
| High (n = 211) |  |  |  |  |  |
| n | 82 | 46 | 83 |  |  |
| Age-adjusted HR (95% CI)^†^ | 1 (referent) | 1.03 (0.71-1.49) | 1.64 (1.21-2.23) | 0.001 |  |
| Multivariable HR (95% CI)^†‡^ | 1 (referent) | 1.07 (0.74-1.54) | 1.60 (1.17-2.18) | 0.004 |  |
|  |  |  |  |  |  |

* Based on all colorectal cancer cases with available whole-exome sequencing data, tumour mutational burden based on selected cancer-associated genes was categorised into high (≥ 8, the top quartile) and low (< 8, the other quartiles).

^†^ Inverse probability weighting was applied to reduce a potential selection bias due to the differential availability of whole-exome sequencing data (see “Statistical analysis” subsection for details).

^‡^ The multivariable Cox regression model was adjusted for the same set of covariates as Table 2.

^§^ P_trend_ was calculated using a linear trend test and cumulative pack-years smoked (continuous with a ceiling at 50 pack-years).

^¶^ P_heterogeneity_ was calculated using the likelihood ratio test (one degree of freedom) for the heterogeneity of binary subtype-specific associations of cumulative pack-years smoked (continuous with a ceiling at 50 pack-years) in multivariable models.

BWH, the Brigham and Women’s Hospital; CI, confidence interval; DFCI, Dana-Farber Cancer Institute; HR, hazard ratio; TMB, tumour mutational burden.

Supplementary Table S7. Smoking status and colorectal cancer incidence, overall and by exome-wide tumour mutational burden or neoantigen loads

|  | Smoking status | | |  |  |
| --- | --- | --- | --- | --- | --- |
|  | Never | Former | Current | P_trend_^§^ | P_heterogeneity_^¶^ |
| Person-years | 1,609,012 | 1,395,090 | 437,566 |  |  |
|  |  |  |  |  |  |
| **All colorectal cancer** (n = 749) |  |  |  |  |  |
| n | 316 | 362 | 71 |  |  |
| Age-adjusted HR (95% CI)^†^ | 1 (referent) | 1.17 (1.01-1.37) | 1.28 (0.98-1.66) | 0.017 | - |
| Multivariable HR (95% CI)^†‡^ | 1 (referent) | 1.18 (1.01-1.38) | 1.18 (0.90-1.55) | 0.049 | - |
|  |  |  |  |  |  |
| **Exome-wide tumour mutational burden*** | |  |  |  | 0.003 |
| Low (n = 651) |  |  |  |  |  |
| n | 286 | 309 | 56 |  |  |
| Age-adjusted HR (95% CI)^†^ | 1 (referent) | 1.12 (0.95-1.31) | 1.08 (0.81-1.45) | 0.25 |  |
| Multivariable HR (95% CI)^†‡^ | 1 (referent) | 1.14 (0.96-1.34) | 1.02 (0.76-1.38) | 0.33 |  |
| High (n = 98) |  |  |  |  |  |
| n | 30 | 53 | 15 |  |  |
| Age-adjusted HR (95% CI)^†^ | 1 (referent) | 1.87 (1.19-2.95) | 2.86 (1.51-5.42) | < 0.001 |  |
| Multivariable HR (95% CI)^†‡^ | 1 (referent) | 1.91 (1.21-3.01) | 2.72 (1.43-5.18) | < 0.001 |  |
|  |  |  |  |  |  |
| **Neoantigen loads*** |  |  |  |  | 0.18 |
| Low (n = 561) |  |  |  |  |  |
| n | 242 | 266 | 53 |  |  |
| Age-adjusted HR (95% CI)^†^ | 1 (referent) | 1.13 (0.95-1.35) | 1.17 (0.86-1.59) | 0.14 |  |
| Multivariable HR (95% CI)^†‡^ | 1 (referent) | 1.15 (0.96-1.37) | 1.11 (0.81-1.51) | 0.20 |  |
| High (n = 188) |  |  |  |  |  |
| n | 74 | 96 | 18 |  |  |
| Age-adjusted HR (95% CI)^†^ | 1 (referent) | 1.38 (1.01-1.87) | 1.56 (0.93-2.63) | 0.019 |  |
| Multivariable HR (95% CI)^†‡^ | 1 (referent) | 1.42 (1.05-1.93) | 1.47 (0.87-2.49) | 0.021 |  |
|  |  |  |  |  |  |

* e-TMB was categorised into high (≥ 10 per megabase) and low (< 10 per megabase). Based on all colorectal cancer cases with available whole-exome sequencing data, neoantigen loads were categorised into high (≥ 326 per exome, the top quartile) and low (< 326 per exome, the other quartiles).

^†^ Inverse probability weighting was applied to reduce a potential selection bias due to the differential availability of whole-exome sequencing data (see “Statistical analysis” subsection for details).

^‡^ The multivariable Cox regression model was adjusted for the same set of covariates as Table 2.

^§^ P_trend_ was calculated using a linear trend test and ordinal categories of smoking status (never, former, and current).

^¶^ P_heterogeneity_ was calculated using the likelihood ratio test (one degree of freedom) for the heterogeneity of binary subtype-specific associations of smoking status (never, former, and current) in multivariable models.

CI, confidence interval; e-TMB, exome-wide tumour mutational burden; HR, hazard ratio.

Supplementary table S8. Duration of smoking cessation and colorectal cancer incidence, overall and by exome-wide tumour mutational burden or neoantigen loads

|  | Duration of smoking cessation, years | | |  |  |
| --- | --- | --- | --- | --- | --- |
|  | 0 | 1-9 | ≥ 10 | P_trend_^§^ | P_heterogeneity_^¶^ |
| Person-years | 437,566 | 317,375 | 1,063,700 |  |  |
|  |  |  |  |  |  |
| **All colorectal cancer** (n = 429) |  |  |  |  |  |
| n | 71 | 75 | 283 |  |  |
| Age-adjusted HR (95% CI)^†^ | 1 (referent) | 1.06 (0.88-1.29) | 0.68 (0.43-1.10) | 0.022 | - |
| Multivariable HR (95% CI)^†‡^ | 1 (referent) | 1.10 (0.91-1.34) | 0.69 (0.43-1.10) | 0.12 | - |
|  |  |  |  |  |  |
| **Exome-wide tumour mutational burden*** | |  |  |  | 0.001 |
| Low (n = 361) |  |  |  |  |  |
| n | 56 | 55 | 250 |  |  |
| Age-adjusted HR (95% CI)^†^ | 1 (referent) | 1.12 (0.89-1.41) | 0.72 (0.41-1.25) | 0.34 |  |
| Multivariable HR (95% CI)^†‡^ | 1 (referent) | 1.15 (0.92-1.45) | 0.72 (0.42-1.25) | 0.71 |  |
| High (n = 68) |  |  |  |  |  |
| n | 15 | 20 | 33 |  |  |
| Age-adjusted HR (95% CI)^†^ | 1 (referent) | 1.15 (0.80-1.66) | 0.25 (0.10-0.63) | < 0.001 |  |
| Multivariable HR (95% CI)^†‡^ | 1 (referent) | 1.19 (0.82-1.72) | 0.25 (0.10-0.63) | < 0.001 |  |
|  |  |  |  |  |  |
| **Neoantigen loads*** |  |  |  |  | 0.010 |
| Low (n = 316) |  |  |  |  |  |
| n | 53 | 48 | 215 |  |  |
| Age-adjusted HR (95% CI)^†^ | 1 (referent) | 1.08 (0.85-1.37) | 0.76 (0.42-1.38) | 0.43 |  |
| Multivariable HR (95% CI)^†‡^ | 1 (referent) | 1.11 (0.87-1.41) | 0.76 (0.42-1.38) | 0.78 |  |
| High (n = 113) |  |  |  |  |  |
| n | 18 | 27 | 68 |  |  |
| Age-adjusted HR (95% CI)^†^ | 1 (referent) | 1.25 (0.90-1.72) | 0.32 (0.15-0.71) | < 0.001 |  |
| Multivariable HR (95% CI)^†‡^ | 1 (referent) | 1.28 (0.92-1.78) | 0.33 (0.15-0.72) | 0.001 |  |
|  |  |  |  |  |  |

* e-TMB was categorised into high (≥ 10 per megabase) and low (< 10 per megabase). Based on all colorectal cancer cases with available whole-exome sequencing data, neoantigen loads were categorised into high (≥ 326 per exome, the top quartile) and low (< 326 per exome, the other quartiles).

^†^ Inverse probability weighting was applied to reduce a potential selection bias due to the differential availability of whole-exome sequencing data (see “Statistical analysis” subsection for details).

^‡^ The multivariable Cox regression model was adjusted for the same set of covariates as Table 2.

^§^ P_trend_ was calculated using a linear trend test and duration of smoking cessation (continuous with a ceiling at 40 years).

^¶^ P_heterogeneity_ was calculated using the likelihood ratio test (one degree of freedom) for the heterogeneity of binary subtype-specific associations of duration of smoking cessation (continuous with a ceiling at 40 years) in multivariable models.

CI, confidence interval; e-TMB, exome-wide tumour mutational burden; HR, hazard ratio.

Supplementary table S9. Cumulative pack-years smoked and colorectal cancer incidence by exome-wide tumour mutational burden or neoantigen loads in the strata of CpG island methylator phenotype (CIMP), *BRAF* mutation, or lymphocytic reaction status

|  | Cumulative pack-years smoked | | |  |
| --- | --- | --- | --- | --- |
|  | 0 | 1-19 | ≥ 20 | P_trend_^§^ |
| **CIMP-low/negative** |  |  |  |  |
| **Exome-wide tumour mutational burden*** | |  |  |  |
| Low (n = 521) | 221 | 144 | 156 |  |
| Age-adjusted HR (95% CI)^†^ | 1 (referent) | 1.21 (0.98-1.50) | 1.09 (0.89-1.34) | 0.45 |
| Multivariable HR (95% CI)^†‡^ | 1 (referent) | 1.25 (1.01-1.55) | 1.05 (0.85-1.31) | 0.85 |
| High (n = 28) | 12 | 5 | 11 |  |
| Age-adjusted HR (95% CI)^†^ | 1 (referent) | 0.76 (0.26-2.24) | 1.47 (0.62-3.49) | 0.44 |
| Multivariable HR (95% CI)^†‡^ | 1 (referent) | 0.79 (0.27-2.33) | 1.43 (0.60-3.39) | 0.51 |
|  |  |  |  |  |
| **Neoantigen loads*** |  |  |  |  |
| Low (n = 480) | 201 | 138 | 141 |  |
| Age-adjusted HR (95% CI)^†^ | 1 (referent) | 1.28 (1.03-1.59) | 1.08 (0.87-1.34) | 0.43 |
| Multivariable HR (95% CI)^†‡^ | 1 (referent) | 1.32 (1.06-1.64) | 1.04 (0.83-1.30) | 0.82 |
| High (n = 69) | 32 | 11 | 26 |  |
| Age-adjusted HR (95% CI)^†^ | 1 (referent) | 0.62 (0.31-1.25) | 1.28 (0.76-2.18) | 0.61 |
| Multivariable HR (95% CI)^†‡^ | 1 (referent) | 0.66 (0.33-1.33) | 1.27 (0.75-2.15) | 0.72 |
|  |  |  |  |  |
| **CIMP-high** |  |  |  |  |
| **Exome-wide tumour mutational burden*** | |  |  |  |
| Low (n = 60) | 29 | 10 | 21 |  |
| Age-adjusted HR (95% CI)^†^ | 1 (referent) | 0.66 (0.32-1.36) | 1.14 (0.64-2.03) | 0.61 |
| Multivariable HR (95% CI)^†‡^ | 1 (referent) | 0.66 (0.32-1.36) | 1.16 (0.65-2.09) | 0.58 |
| High (n = 66) | 18 | 13 | 35 |  |
| Age-adjusted HR (95% CI)^†^ | 1 (referent) | 1.34 (0.65-2.78) | 3.06 (1.73-5.42) | < 0.001 |
| Multivariable HR (95% CI)^†‡^ | 1 (referent) | 1.39 (0.67-2.86) | 3.15 (1.75-5.67) | < 0.001 |
|  |  |  |  |  |
| **Neoantigen loads*** |  |  |  |  |
| Low (n = 23) | 13 | 5 | 5 |  |
| Age-adjusted HR (95% CI)^†^ | 1 (referent) | 0.64 (0.23-1.80) | 0.56 (0.19-1.66) | 0.73 |
| Multivariable HR (95% CI)^†‡^ | 1 (referent) | 0.64 (0.23-1.77) | 0.58 (0.19-1.72) | 0.76 |
| High (n = 103) | 34 | 18 | 51 |  |
| Age-adjusted HR (95% CI)^†^ | 1 (referent) | 1.04 (0.58-1.86) | 2.41 (1.56-3.74) | < 0.001 |
| Multivariable HR (95% CI)^†‡^ | 1 (referent) | 1.07 (0.60-1.91) | 2.48 (1.57-3.91) | < 0.001 |
|  |  |  |  |  |
| ***BRAF* wild-type** |  |  |  |  |
| **Exome-wide tumour mutational burden*** | |  |  |  |
| Low (n = 542) | 234 | 149 | 159 |  |
| Age-adjusted HR (95% CI)^†^ | 1 (referent) | 1.20 (0.97-1.47) | 1.06 (0.86-1.30) | 0.64 |
| Multivariable HR (95% CI)^†‡^ | 1 (referent) | 1.23 (1.00-1.52) | 1.02 (0.83-1.26) | 0.93 |
| High (n = 49) | 18 | 11 | 20 |  |
| Age-adjusted HR (95% CI)^†^ | 1 (referent) | 1.22 (0.57-2.63) | 1.75 (0.91-3.35) | 0.041 |
| Multivariable HR (95% CI)^†‡^ | 1 (referent) | 1.26 (0.59-2.70) | 1.69 (0.88-3.23) | 0.061 |
|  |  |  |  |  |
|  |  |  |  |  |
|  |  |  |  |  |
| **Neoantigen loads*** |  |  |  |  |
| Low (n = 497) | 209 | 143 | 145 |  |
| Age-adjusted HR (95% CI)^†^ | 1 (referent) | 1.28 (1.04-1.59) | 1.07 (0.87-1.33) | 0.48 |
| Multivariable HR (95% CI)^†‡^ | 1 (referent) | 1.32 (1.06-1.64) | 1.03 (0.82-1.29) | 0.90 |
| High (n = 94) | 43 | 17 | 34 |  |
| Age-adjusted HR (95% CI)^†^ | 1 (referent) | 0.76 (0.43-1.35) | 1.27 (0.81-2.01) | 0.30 |
| Multivariable HR (95% CI)^†‡^ | 1 (referent) | 0.81 (0.46-1.43) | 1.26 (0.80-1.99) | 0.36 |
|  |  |  |  |  |
| ***BRAF* mutant** |  |  |  |  |
| **Exome-wide tumour mutational burden*** | |  |  |  |
| Low (n = 71) | 33 | 13 | 25 |  |
| Age-adjusted HR (95% CI)^†^ | 1 (referent) | 0.75 (0.40-1.41) | 1.14 (0.66-1.95) | 0.53 |
| Multivariable HR (95% CI)^†‡^ | 1 (referent) | 0.75 (0.41-1.40) | 1.14 (0.66-1.98) | 0.51 |
| High (n = 47) | 12 | 9 | 26 |  |
| Age-adjusted HR (95% CI)^†^ | 1 (referent) | 1.30 (0.54-3.15) | 3.47 (1.74-6.94) | < 0.001 |
| Multivariable HR (95% CI)^†‡^ | 1 (referent) | 1.32 (0.54-3.22) | 3.53 (1.73-7.20) | < 0.001 |
|  |  |  |  |  |
| **Neoantigen loads*** |  |  |  |  |
| Low (n = 35) | 20 | 7 | 8 |  |
| Age-adjusted HR (95% CI)^†^ | 1 (referent) | 0.59 (0.26-1.35) | 0.54 (0.22-1.29) | 0.48 |
| Multivariable HR (95% CI)^†‡^ | 1 (referent) | 0.59 (0.26-1.33) | 0.55 (0.23-1.33) | 0.52 |
| High (n = 83) | 25 | 15 | 43 |  |
| Age-adjusted HR (95% CI)^†^ | 1 (referent) | 1.13 (0.59-2.16) | 2.77 (1.69-4.56) | < 0.001 |
| Multivariable HR (95% CI)^†‡^ | 1 (referent) | 1.15 (0.60-2.19) | 2.80 (1.68-4.68) | < 0.001 |
|  |  |  |  |  |
| **Tumour-infiltrating lymphocytes, absent**^¶^ | |  |  |  |
| **Exome-wide tumour mutational burden*** | |  |  |  |
| Low (n = 501) | 234 | 127 | 140 |  |
| Age-adjusted HR (95% CI)^†^ | 1 (referent) | 1.03 (0.83-1.28) | 0.91 (0.74-1.13) | 0.63 |
| Multivariable HR (95% CI)^†‡^ | 1 (referent) | 1.08 (0.87-1.34) | 0.88 (0.71-1.10) | 0.34 |
| High (n = 25) | 3 | 8 | 14 |  |
| Age-adjusted HR (95% CI)^†^ | 1 (referent) | 4.20 (1.13-15.6) | 7.27 (1.97-26.8) | < 0.001 |
| Multivariable HR (95% CI)^†‡^ | 1 (referent) | 4.37 (1.18-16.1) | 6.95 (1.91-25.3) | < 0.001 |
|  |  |  |  |  |
| **Neoantigen loads*** |  |  |  |  |
| Low (n = 455) | 207 | 119 | 129 |  |
| Age-adjusted HR (95% CI)^†^ | 1 (referent) | 1.09 (0.87-1.37) | 0.95 (0.76-1.18) | 0.92 |
| Multivariable HR (95% CI)^†‡^ | 1 (referent) | 1.13 (0.90-1.42) | 0.91 (0.73-1.15) | 0.56 |
| High (n = 71) | 30 | 16 | 25 |  |
| Age-adjusted HR (95% CI)^†^ | 1 (referent) | 0.95 (0.52-1.75) | 1.34 (0.77-2.32) | 0.16 |
| Multivariable HR (95% CI)^†‡^ | 1 (referent) | 1.02 (0.56-1.85) | 1.31 (0.76-2.26) | 0.20 |
|  |  |  |  |  |
| **Tumour-infiltrating lymphocytes, present**^¶^ | |  |  |  |
| **Exome-wide tumour mutational burden*** | |  |  |  |
| Low (n = 139) | 47 | 40 | 52 |  |
| Age-adjusted HR (95% CI)^†^ | 1 (referent) | 1.56 (1.02-2.39) | 1.74 (1.17-2.59) | 0.021 |
| Multivariable HR (95% CI)^†‡^ | 1 (referent) | 1.57 (1.02-2.41) | 1.75 (1.16-2.65) | 0.028 |
| High (n = 71) | 26 | 12 | 33 |  |
| Age-adjusted HR (95% CI)^†^ | 1 (referent) | 0.92 (0.45-1.87) | 2.07 (1.23-3.49) | 0.005 |
| Multivariable HR (95% CI)^†‡^ | 1 (referent) | 0.93 (0.46-1.88) | 2.10 (1.24-3.57) | 0.005 |
|  |  |  |  |  |
|  |  |  |  |  |
| **Neoantigen loads*** |  |  |  |  |
| Low (n = 95) | 30 | 33 | 32 |  |
| Age-adjusted HR (95% CI)^†^ | 1 (referent) | 1.94 (1.17-3.19) | 1.63 (0.98-2.70) | 0.075 |
| Multivariable HR (95% CI)^†‡^ | 1 (referent) | 1.94 (1.17-3.21) | 1.64 (0.97-2.75) | 0.081 |
| High (n = 115) | 43 | 19 | 53 |  |
| Age-adjusted HR (95% CI)^†^ | 1 (referent) | 0.89 (0.51-1.55) | 2.03 (1.36-3.04) | 0.002 |
| Multivariable HR (95% CI)^†‡^ | 1 (referent) | 0.90 (0.52-1.57) | 2.05 (1.35-3.11) | 0.002 |
|  |  |  |  |  |
| **Intratumoural periglandular reaction, low**^¶^ | |  |  |  |
| **Exome-wide tumour mutational burden*** | |  |  |  |
| Low (n = 558) | 247 | 150 | 161 |  |
| Age-adjusted HR (95% CI)^†^ | 1 (referent) | 1.14 (0.93-1.40) | 0.99 (0.81-1.21) | 0.76 |
| Multivariable HR (95% CI)^†‡^ | 1 (referent) | 1.19 (0.97-1.47) | 0.98 (0.79-1.21) | > 0.99 |
| High (n = 66) | 19 | 16 | 31 |  |
| Age-adjusted HR (95% CI)^†^ | 1 (referent) | 1.52 (0.77-3.03) | 2.64 (1.49-4.69) | < 0.001 |
| Multivariable HR (95% CI)^†‡^ | 1 (referent) | 1.59 (0.80-3.16) | 2.61 (1.47-4.62) | < 0.001 |
|  |  |  |  |  |
| **Neoantigen loads*** |  |  |  |  |
| Low (n = 495) | 215 | 137 | 143 |  |
| Age-adjusted HR (95% CI)^†^ | 1 (referent) | 1.20 (0.97-1.48) | 1.01 (0.81-1.25) | 0.58 |
| Multivariable HR (95% CI)^†‡^ | 1 (referent) | 1.24 (1.00-1.55) | 0.99 (0.80-1.24) | 0.80 |
| High (n = 129) | 51 | 29 | 49 |  |
| Age-adjusted HR (95% CI)^†^ | 1 (referent) | 1.06 (0.67-1.68) | 1.55 (1.04-2.32) | 0.041 |
| Multivariable HR (95% CI)^†‡^ | 1 (referent) | 1.12 (0.71-1.78) | 1.55 (1.04-2.31) | 0.056 |
|  |  |  |  |  |
| **Intratumoural periglandular reaction, high**^¶^ | |  |  |  |
| **Exome-wide tumour mutational burden*** | |  |  |  |
| Low (n = 84) | 35 | 17 | 32 |  |
| Age-adjusted HR (95% CI)^†^ | 1 (referent) | 0.92 (0.52-1.66) | 1.51 (0.94-2.43) | 0.26 |
| Multivariable HR (95% CI)^†‡^ | 1 (referent) | 0.90 (0.50-1.61) | 1.40 (0.86-2.28) | 0.47 |
| High (n = 30) | 10 | 4 | 16 |  |
| Age-adjusted HR (95% CI)^†^ | 1 (referent) | 0.80 (0.25-2.57) | 2.56 (1.12-5.88) | 0.007 |
| Multivariable HR (95% CI)^†‡^ | 1 (referent) | 0.78 (0.24-2.51) | 2.38 (1.03-5.55) | 0.014 |
|  |  |  |  |  |
| **Neoantigen loads*** |  |  |  |  |
| Low (n = 57) | 23 | 15 | 19 |  |
| Age-adjusted HR (95% CI)^†^ | 1 (referent) | 1.18 (0.61-2.26) | 1.32 (0.71-2.43) | 0.61 |
| Multivariable HR (95% CI)^†‡^ | 1 (referent) | 1.14 (0.59-2.20) | 1.22 (0.65-2.29) | 0.84 |
| High (n = 57) | 22 | 6 | 29 |  |
| Age-adjusted HR (95% CI)^†^ | 1 (referent) | 0.57 (0.23-1.43) | 2.23 (1.28-3.89) | 0.003 |
| Multivariable HR (95% CI)^†‡^ | 1 (referent) | 0.56 (0.23-1.40) | 2.07 (1.17-3.65) | 0.009 |
|  |  |  |  |  |
| **Peritumoural lymphocytic reaction, low**^¶^ | |  |  |  |
| **Exome-wide tumour mutational burden*** | |  |  |  |
| Low (n = 526) | 237 | 138 | 151 |  |
| Age-adjusted HR (95% CI)^†^ | 1 (referent) | 1.10 (0.89-1.36) | 0.96 (0.78-1.18) | 0.98 |
| Multivariable HR (95% CI)^†‡^ | 1 (referent) | 1.16 (0.93-1.43) | 0.95 (0.77-1.18) | 0.77 |
| High (n = 61) | 18 | 13 | 30 |  |
| Age-adjusted HR (95% CI)^†^ | 1 (referent) | 1.30 (0.62-2.73) | 2.65 (1.47-4.77) | < 0.001 |
| Multivariable HR (95% CI)^†‡^ | 1 (referent) | 1.37 (0.65-2.86) | 2.64 (1.47-4.74) | 0.001 |
|  |  |  |  |  |
|  |  |  |  |  |
| **Neoantigen loads*** |  |  |  |  |
| Low (n = 464) | 203 | 126 | 135 |  |
| Age-adjusted HR (95% CI)^†^ | 1 (referent) | 1.17 (0.94-1.46) | 1.00 (0.80-1.24) | 0.63 |
| Multivariable HR (95% CI)^†‡^ | 1 (referent) | 1.22 (0.98-1.53) | 0.99 (0.79-1.24) | 0.83 |
| High (n = 123) | 52 | 25 | 46 |  |
| Age-adjusted HR (95% CI)^†^ | 1 (referent) | 0.91 (0.56-1.48) | 1.41 (0.95-2.11) | 0.15 |
| Multivariable HR (95% CI)^†‡^ | 1 (referent) | 0.97 (0.60-1.57) | 1.42 (0.95-2.12) | 0.17 |
|  |  |  |  |  |
| **Peritumoural lymphocytic reaction, high**^¶^ | |  |  |  |
| **Exome-wide tumour mutational burden*** | |  |  |  |
| Low (n = 115) | 45 | 28 | 42 |  |
| Age-adjusted HR (95% CI)^†^ | 1 (referent) | 1.14 (0.71-1.84) | 1.56 (1.02-2.38) | 0.091 |
| Multivariable HR (95% CI)^†‡^ | 1 (referent) | 1.13 (0.70-1.81) | 1.46 (0.94-2.28) | 0.22 |
| High (n = 35) | 11 | 7 | 17 |  |
| Age-adjusted HR (95% CI)^†^ | 1 (referent) | 1.23 (0.47-3.18) | 2.57 (1.16-5.66) | 0.004 |
| Multivariable HR (95% CI)^†‡^ | 1 (referent) | 1.21 (0.47-3.13) | 2.42 (1.09-5.37) | 0.008 |
|  |  |  |  |  |
| **Neoantigen loads*** |  |  |  |  |
| Low (n = 87) | 35 | 25 | 27 |  |
| Age-adjusted HR (95% CI)^†^ | 1 (referent) | 1.28 (0.77-2.15) | 1.26 (0.76-2.10) | 0.53 |
| Multivariable HR (95% CI)^†‡^ | 1 (referent) | 1.25 (0.75-2.09) | 1.17 (0.69-1.95) | 0.79 |
| High (n = 63) | 21 | 10 | 32 |  |
| Age-adjusted HR (95% CI)^†^ | 1 (referent) | 0.93 (0.44-2.00) | 2.61 (1.50-4.56) | < 0.001 |
| Multivariable HR (95% CI)^†‡^ | 1 (referent) | 0.93 (0.43-2.00) | 2.48 (1.41-4.37) | < 0.001 |
|  |  |  |  |  |
| **Crohn's-like lymphoid reaction, absent**^¶^ | |  |  |  |
| **Exome-wide tumour mutational burden*** | |  |  |  |
| Low (n = 451) | 214 | 119 | 118 |  |
| Age-adjusted HR (95% CI)^†^ | 1 (referent) | 1.06 (0.85-1.33) | 0.86 (0.69-1.08) | 0.45 |
| Multivariable HR (95% CI)^†‡^ | 1 (referent) | 1.10 (0.88-1.38) | 0.84 (0.66-1.07) | 0.30 |
| High (n = 33) | 5 | 9 | 19 |  |
| Age-adjusted HR (95% CI)^†^ | 1 (referent) | 3.11 (1.03-9.40) | 5.85 (2.15-15.9) | < 0.001 |
| Multivariable HR (95% CI)^†‡^ | 1 (referent) | 3.27 (1.08-9.87) | 5.70 (2.11-15.4) | < 0.001 |
|  |  |  |  |  |
| **Neoantigen loads*** |  |  |  |  |
| Low (n = 402) | 186 | 111 | 105 |  |
| Age-adjusted HR (95% CI)^†^ | 1 (referent) | 1.14 (0.90-1.44) | 0.89 (0.69-1.13) | 0.68 |
| Multivariable HR (95% CI)^†‡^ | 1 (referent) | 1.17 (0.93-1.49) | 0.86 (0.67-1.11) | 0.49 |
| High (n = 82) | 33 | 17 | 32 |  |
| Age-adjusted HR (95% CI)^†^ | 1 (referent) | 0.98 (0.55-1.77) | 1.51 (0.93-2.47) | 0.070 |
| Multivariable HR (95% CI)^†‡^ | 1 (referent) | 1.06 (0.59-1.90) | 1.51 (0.92-2.45) | 0.091 |
|  |  |  |  |  |
| **Crohn's-like lymphoid reaction, present**^¶^ | |  |  |  |
| **Exome-wide tumour mutational burden*** | |  |  |  |
| Low (n = 105) | 35 | 25 | 45 |  |
| Age-adjusted HR (95% CI)^†^ | 1 (referent) | 1.33 (0.79-2.25) | 2.00 (1.30-3.07) | 0.002 |
| Multivariable HR (95% CI)^†‡^ | 1 (referent) | 1.37 (0.80-2.32) | 1.99 (1.28-3.11) | 0.003 |
| High (n = 52) | 18 | 9 | 25 |  |
| Age-adjusted HR (95% CI)^†^ | 1 (referent) | 0.96 (0.42-2.17) | 2.33 (1.26-4.29) | 0.002 |
| Multivariable HR (95% CI)^†‡^ | 1 (referent) | 0.99 (0.44-2.23) | 2.32 (1.25-4.33) | 0.002 |
|  |  |  |  |  |
|  |  |  |  |  |
| **Neoantigen loads*** |  |  |  |  |
| Low (n = 74) | 24 | 20 | 30 |  |
| Age-adjusted HR (95% CI)^†^ | 1 (referent) | 1.49 (0.81-2.74) | 1.79 (1.06-3.02) | 0.012 |
| Multivariable HR (95% CI)^†‡^ | 1 (referent) | 1.52 (0.82-2.81) | 1.79 (1.05-3.06) | 0.017 |
| High (n = 83) | 29 | 14 | 40 |  |
| Age-adjusted HR (95% CI)^†^ | 1 (referent) | 0.95 (0.49-1.81) | 2.39 (1.49-3.85) | < 0.001 |
| Multivariable HR (95% CI)^†‡^ | 1 (referent) | 0.98 (0.51-1.87) | 2.38 (1.46-3.90) | < 0.001 |
|  |  |  |  |  |

* e-TMB was categorised into high (≥ 10 per megabase) and low (< 10 per megabase). Based on all colorectal cancer cases with available whole-exome sequencing data, neoantigen loads were categorised into high (≥ 326 per exome, the top quartile) and low (< 326 per exome, the other quartiles).

^†^ Inverse probability weighting was applied to reduce a potential selection bias due to the differential availability of whole-exome sequencing data (see “Statistical analysis” subsection for details).

^‡^ The multivariable Cox regression model was adjusted for the same set of covariates as Table 2.

^§^ P_trend_ was calculated using a linear trend test and cumulative pack-years smoked (continuous with a ceiling at 50 pack-years).

^¶^ Each lymphocytic reaction pattern was categorised as the most common level or lower-level reaction (*i.e.*, negative/low for tumour-infiltrating lymphocytes and Crohn’s-like lymphoid reaction; and negative/low to intermediate for intratumoural periglandular reaction and peritumoural lymphocytic reaction) vs. higher-level reaction.

CI, confidence interval; CIMP, CpG island methylator phenotype; e-TMB, exome-wide tumour mutational burden; HR, hazard ratio.
